# Brother of the regulator of the imprinted site (BORIS) variant subfamily 6 is involved in cervical cancer stemness and can be a target of immunotherapy

**DOI:** 10.18632/oncotarget.7165

**Published:** 2016-02-03

**Authors:** Takuya Asano, Yoshihiko Hirohashi, Toshihiko Torigoe, Tasuku Mariya, Ryota Horibe, Takafumi Kuroda, Yuta Tabuchi, Hiroshi Saijo, Kazuyo Yasuda, Masahito Mizuuchi, Akari Takahashi, Hiroko Asanuma, Tadashi Hasegawa, Tsuyoshi Saito, Noriyuki Sato

**Affiliations:** ^1^ Departments of Pathology, Sapporo Medical University School of Medicine, Sapporo, Japan; ^2^ Obsterics and Gynecology, Sapporo Medical University School of Medicine, Sapporo, Japan; ^3^ Respiratory Medicine and Allergology, Sapporo Medical University School of Medicine, Sapporo, Japan; ^4^ Surgical Pathology, Sapporo Medical University School of Medicine, Sapporo, Japan

**Keywords:** cancer stem cells, cervical cancer, CTL, BORIS, peptide vaccine

## Abstract

Cervical cancer is a major cause of cancer death in females worldwide. Cervical cancer stem-like cells (CSCs)/cancer-initiating cells (CICs) are resistant to conventional radiotherapy and chemotherapy, and CSCs/CICs are thought to be responsible for recurrence. Eradication of CSCs/CICs is thus essential to cure cervical cancer. In this study, we isolated cervical CSCs/CICs by sphere culture, and we identified a cancer testis (CT) antigen, CTCFL/BORIS, that is expressed in cervical CSCs/CICs. BORIS has 23 mRNA isoform variants classified by 6 subfamilies (sfs), and they encode 17 different BORIS peptides. BORIS sf1 and sf4 are expressed in both CSCs/CICs and non-CSCs/CICs, whereas BORIS sf6 is expressed only in CSCs/CICs. Overexpression of BORIS sf6 in cervical cancer cells increased sphere formation and tumor-initiating ability compared with those in control cells, whereas overexpression of BORIS sf1 and BORIS sf4 resulted in only slight increases. Thus, BORIS sf6 is a cervical CSC/CIC-specific subfamily and has a role in the maintenance of cervical CSCs/CICs. BORIS sf6 contains a specific c-terminal domain (C34), and we identified a human leukocyte antigen (HLA)-A2-restricted antigenic peptide, BORIS C34_24(9) encoded by BORIS sf6. A BORIS C34_24(9)-specific cytotoxic T cell (CTL) clone showed cytotoxicity for BORIS sf6-overexpressing cervical cancer cells. Furthermore, the CTL clone significantly suppressed sphere formation of CaSki cells. Taken together, the results indicate that the CT antigen BORIS sf6 is specifically expressed in cervical CSCs/CICs, that BORIS sf6 has a role in the maintenance of CSCs/CICs, and that BORIS C34_24(9) peptide is a promising candidate for cervical CSC/CIC-targeting immunotherapy.

## INTRODUCTION

Cervical cancer is the third most common cancer and the fourth leading cause of cancer death in females worldwide, accounting for 529,800 new cases and 275,100 deaths among females in 2008 [1]. Cytology-based screening has decreased the number of cervical cancer cases and the number of deaths due to cervical cancer [2]. However, treatment of patients with locally advanced cervical cancer (Stage IIb, III and IVa) is difficult. Concurrent chemoradiatiotherapy (CCRT) with platinum agents shows a high response rate and is the standard treatment, but the tumor often relapses and the prognosis of patients with a recurrent tumor is extremely poor due to the limited treatment [3].

Cancer stem-like cells/cancer initiation cells (CSCs/CICs) are defined as a small subpopulation of heterogeneous tumor cells that can renew themselves and have high tumor initiation ability [4, 5]. In a solid tumor, CSCs/CICs were identified as CD44^+^CD24^−/low^ cells isolated from human breast cancer for the first time in 2003 [6], and other studies revealed that CSCs/CICs are resistant to chemotherapy and radiotherapy [7–10]. Cell cycle dormancy, expression of ABC transporters, low level of reactive oxygen species (ROS) and resistance to DNA damage have been reported as mechanisms of treatment resistance. In cervical cancer, it has been reported that CSCs/CICs were isolated from primary cancer or cell lines by the sphere formation assay or ALDFLUOR assay and that they showed high tumor initiation ability and resistance to chemotherapy agents [11–13]. Cervical CSCs/CICs have been isolated using the sphere forming assay and cell surface markers including CD44 [11] and CD49f [12], but potential molecular targets of cervical CSCs/CICs are still not known.

Cancer immunotherapy is an attractive alternative approach to conventional chemotherapy and radiotherapy. Human papilloma virus (HPV) E6 and E7 are oncogenic viral proteins, and they are expressed in cervical cancer from the early stage through to late stage and are therefore attractive targets of immunotherapy. However, the results of clinical trials of therapeutic vaccination targeting E6/E7 for advanced cervical cancer patients were not satisfactory [14, 15]. We previously reported that cytotoxic T cells could recognize CSCs/CICs isolated using the side population (SP) technique and inhibit the tumor initiation ability of colon CSCs/CICs [16]. Furthermore, we identified a novel cancer/testis antigen, DnaJ (Hsp40) homolog, subfamily B, member 8 (DNAJB8), that is preferentially expressed in renal cell carcinoma and colon/rectum CSCs/CICs and has a role in the maintenance of CSCs/CICs [17, 18]. Importantly, Dnajb8 showed a strong anti-tumor effect in a mouse DNA vaccination model [17]. These observations indicate the potency of CSC/CIC-targeting immunotherapy [19, 20]; however, there are no known antigens for cervical CSC/CIC-targeting immunotherapy.

In this study, we isolated cervical CSCs/CICs by sphere culture, and we screened for CSC/CIC-specific genes by using a cDNA microarray to identify cervical CSC/CIC-specific genes. CCCTC-binding factor-like protein (CTCFL), also known as brother of the regulator of the imprinted site (BORIS), was found to be preferentially expressed in cervical CSCs/CICs rather in non-CSCs/CICs, and immunological aspects of BORIS were investigated.

## RESULTS

### Spheres derived from cervical cancer cells exhibit a cancer stem-like cell phenotype

**Figure 1:**
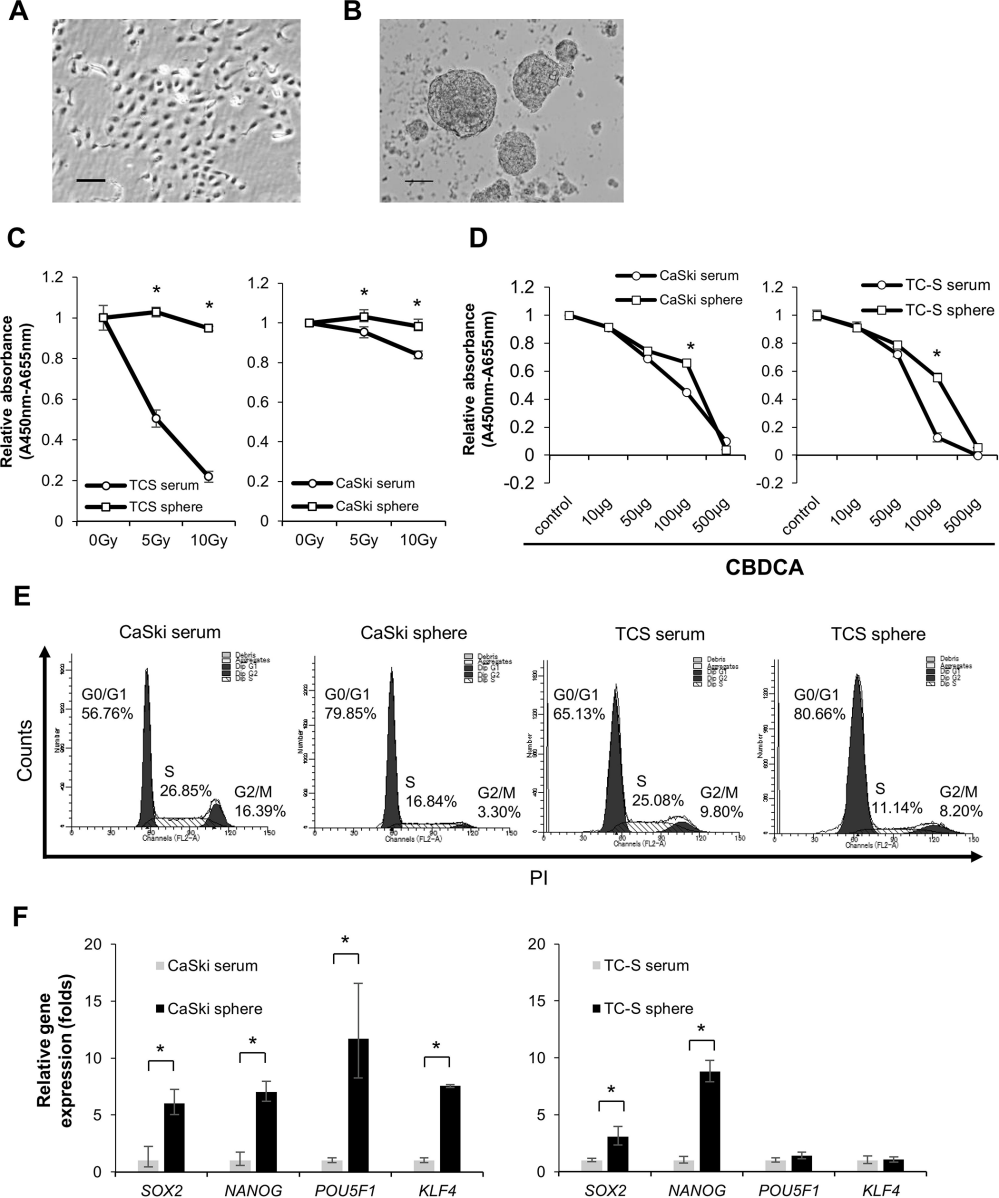
Spheroids have characteristics of CSCs Representative pictures of CaSki cells in a serum cultured condition (**A**) and sphere culture condition (**B**). Magnifications are 40 × and 100 ×, respectively. (**C** and **D**) Resistance to radiotherapy and resistance to chemotherapy. WST-1 analysis showed that spheroid cells derived from cervical cancer cell lines are resistant to irradiation and chemotherapy. Data represent means ± SE. *P < 0.05. (**E**) Cell cycle analysis of spheroids and adherent cultured cells of CaSki and TC-S. (**F**) Expression of Stemness genes in serum cultured cells and spheroids derived from cervical cancer cell lines was evaluated by quantitative RT-PCR. Data represent relative quantity means ± SD. Asterisks indicate statistically significant difference (*p* < 0.01) between serum and sphere cells.

Cervical CSCs/CICs were successfully isolated by sphere culture and ALDEFLUOR assay in previous studies [12, 13]. In this study, we thus cultured cervical cancer line cells CaSki (Figure 1A) and TC-S in a serum-free sphere-forming condition to isolate cervical CSCs/CICs. CaSki and TC-S cells successfully formed spheres (Figure 1B). Sphere-forming cells derived from CaSki cells and TC-S cells showed resistance to radiation and carboplatin (CBDCA) by the WST-1 assay (Figure 1C and 1D). Sphere-forming cells derived from CaSki cells and TC-S cells showed a quiescent cell cycle status compared with cells cultured in serum-containing medium (Figure 1E). Furthermore, sphere-forming cells showed higher expression levels of stem cell-related genes including *SOX2*, *NANOG*, *POU5F1* and *KLF4* (Figure 1F). These results indicate that sphere-forming cells have characteristics of CSCs/CICs, and we used spheres as cervical CSCs/CICs in the following experiments.

### BORIS, a testis-related gene, is expressed in cervical CSCs/CICs, and BORIS expression is related to poorer prognosis of cervical cancer

**Figure 2:**
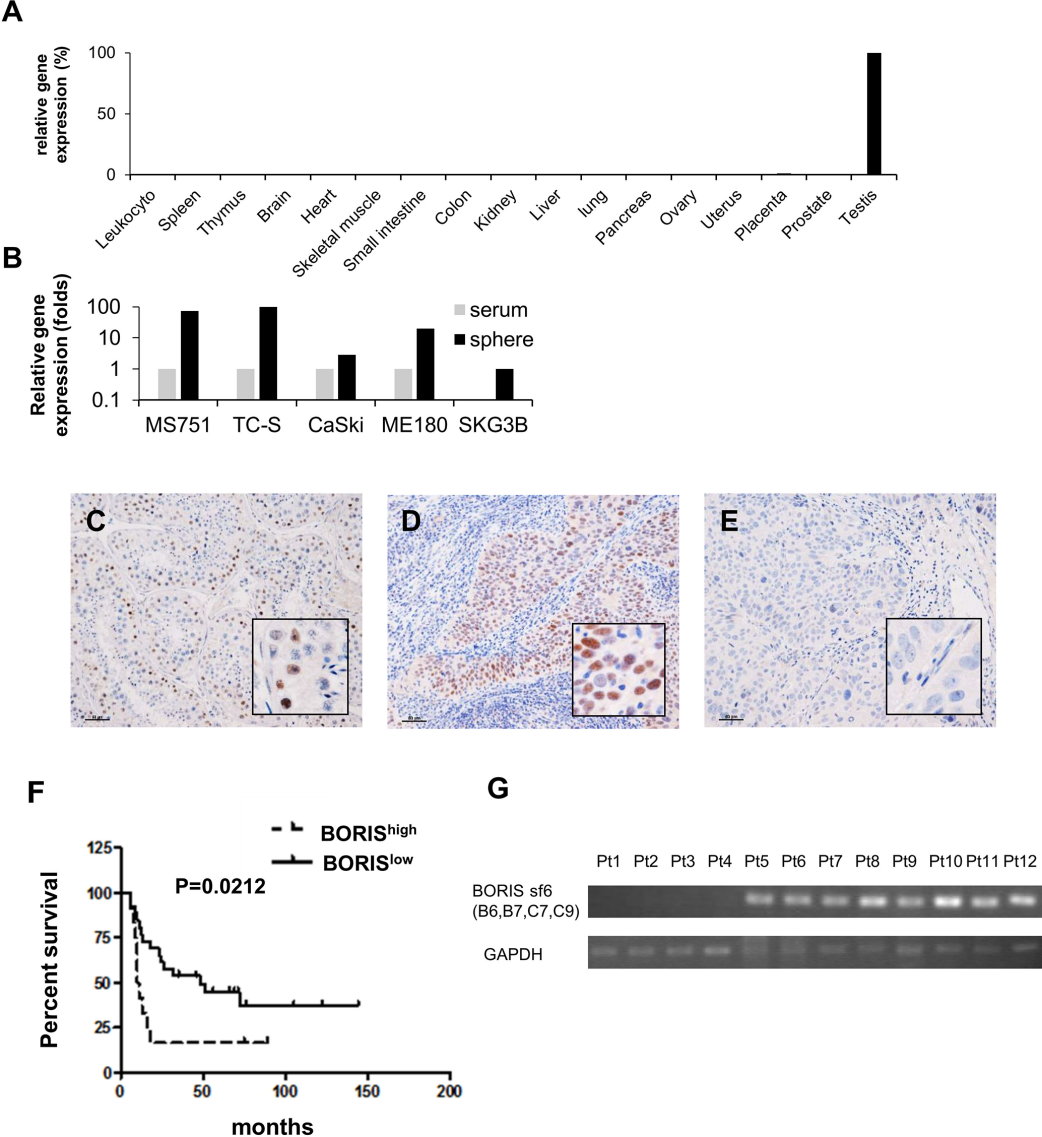
The cancer/testis gene BORIS is preferentially expressed in CSCs and BORIS expression is a marker of poor prognosis for patients with advanced cervical cancer (**A**) Quantitative RT-PCR (qRT-PCR) of BORIS in normal organs. (**B**) QRT-PCR of BORIS in cervical cancer cell lines. Serum: serum culture sells. Sphere: sphere culture cells. Each value is mean relative quantity. Immunohistochemistry of 38 stage III/IV cervical squamous cell carcinoma samples with a BORIS-specific antibody. Immunohistochemical staining of BORIS in a normal testis tissue as a positive control (**C**), a BORIS^high^ case (**D**) and a BORIS^low^ case (**E**). Original magnification is 100 × and the size bar is 50 μm. (**F**) Kaplan-Meier survival estimates were performed according to immunohistochemistry positivity of BORIS. The median survival times of the BORIS^high^ group (*n =* 12) and BORIS^low^ group (*n =* 26) were 10.5 months and 48.0 months, respectively. The log-rank test revealed a significantly worse prognosis for BORIS^high^ cases (*p =* 0.0212). The hazard ratio of BORIS^high^ cases was 2.407 (95% confidence interval: 1.190–8.567). (**G**) BORIS sf6 expression in cervical cancer tissue specimens of BORIS^high^ group by IHC. Tissue samples were analyzed by RT-PCR with BORIS sf6-specific primers. RNA is extracted from formalin-fixed, paraffin-embedded (FFPE) samples.

To explore gene expression profiles of cervical CSCs/CICs, we performed cDNA microarray analysis using total RNAs derived from CaSki sphere-culture cells and CaSki serum-culture cells. Several genes showed higher expression in sphere-culture cells (Supplementary Table S1). Among candidate genes, we focused on *CTCFL/BORIS*, which has been described as a cancer-testis (CT) antigen, since CTCFL/BORIS was preferentially expressed in side population cells derived from colon cancer cell line SW480. [29] BORIS showed specific expression in the testis among normal human organs by quantitative RT-PCR (qRT-PCR) (Figure 2A). BORIS showed preferential expression in sphere-culture cells derived from cervical cancer line cells MS751, TC-S, CaSki ME180 and SKG3B rather than in serum-culture cells (Figure 2B).

To address the expression of BORIS protein in cervical cancer tissues, we performed immunohistochemical staining using an anti-BORIS antibody (*n =* 38). The clinicopathological status of each case is summarized in Table 1. BORIS^high^ expression correlated with older age (*P =* 0.008), but did not with parity, histological type, clinical stage and Initial treatment. Testis tissue was used as a positive control for BORIS staining (Figure 2C). The cases were classified into two groups, BORIS^high^ group, having a BORIS-positive rate of more than 50% (Figure 2D), and BORIS^low^ group, having a BORIS-positive rate of less than 50% (Figure 2E). There were 12 BORIS^high^ cases and 26 BORIS^low^ cases. There was no significant correlation of expression levels of BORIS with age, parity, presence of keratinization, FIGO stage or initial treatment. Kaplan-Meier survival estimates were performed according to immunohistochemistry-positive rates of BORIS. The log-rank test revealed that BORIS^high^ is correlated with poorer prognosis with a significant difference in OS of patients (*P =* 0.0212) (Figure 2F). The median survival times of patients in the BORIS^high^ group and BORIS^low^ group were 10.5 months and 48.0 months, respectively. The hazard ratio of BORIS^high^ cases was 2.407 (95% confidence interval (CI): 1.190–8.567).

**Table 1:** Expression of BORIS and characteristics of cervical SCC patients

	BORIS^high^ ≧ 50% *n* = 12 (%)	BORIS^low^ < 50% *n* = 26 (%)	*p* value
Age	58.9 ± 13.3	45.8 ± 13.5	0.008
parity			
nullpara	4 (33)	7 (27)	0.714
multipara	8 (67)	19 (73)	
			
histological type			
keratinization	4 (33)	9 (35)	1.000
non-keratinization	8 (67)	17 (65)	
Stage			
IIIa	0 (0)	1 (4)	1.000
IIIb	6 (50)	14 (54)	
IVa	2 (17)	4 (15)	
IVb	4 (33)	7 (27)	
Initial treatment			
CCRT	9 (75)	17 (65)	1.000
RT	3 (25)	7 (27)	
NAC+Ope	0 (0)	2 (8)	

Data: Mean ± Standard Deviation for parametric variables, and median (min-max) for non-parametric variables.

SCC: squamous cell carcinoma.

CCRT: concurrent chemoradiation therapy.

RT: radiation therapy.

NAC: neoadjubant chemotherapy.

Ope: operation.

### BORIS subfamily 6 has a role in the maintenance of cervical CSCs/CICs

**Figure 3:**
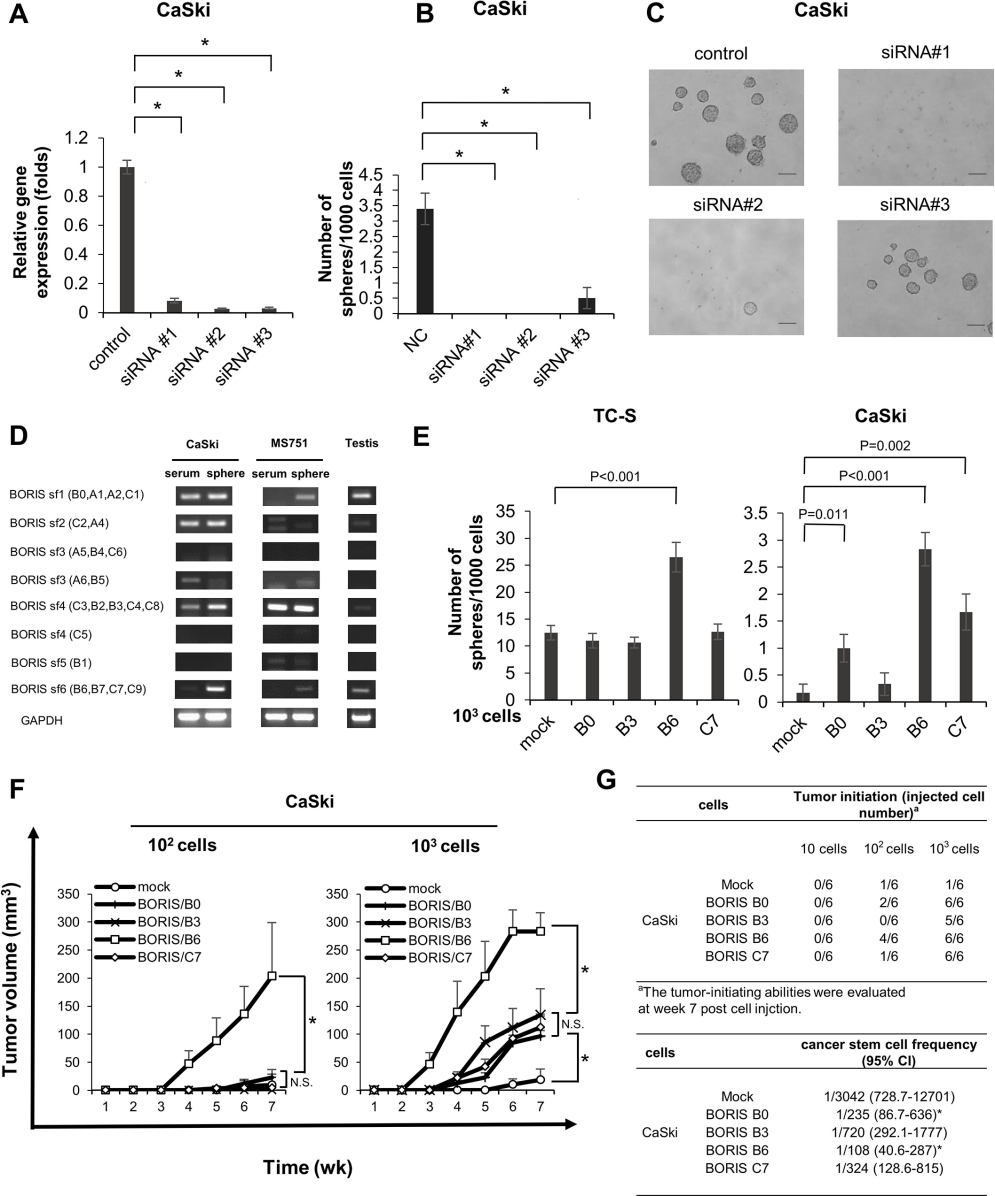
BORIS sf6 is involved in sphere forming ability and cancer initiation ability (**A**) QRT-PCR of CaSki cells transfected with BORIS siRNAs. BORIS mRNA expression in CaSki cells transfected with BORIS-specific siRNA and scrambled siRNA, detected by qRT-PCR analysis. Each value is the mean ± SD of relative quantity (RQ). *P < 0.001. (**B**) Sphere formation of BORIS-knocked-down cells. Each value is the mean ± SD. *P < 0.001, **P = 0.003. (**C**) Images of spheres. Magnification is 10 ×, size bar *=* 100 μm. (**D**) RT-PCR using specific primers for BORIS subfamilies. (**E**) Sphere formation of BORIS variants. Cells with overexpression of each of four different BORIS variants, B0, B3, B6 and C7, were established by using aretroviral vector. Sphere formation assays were performed using stable transfectants. Each value is the mean ± SD. (**F**) Growth curves of tumors derived from CaSki cells transfected with mock and BORIS B0, B3, B6 and C7. Ten, 10^2^ and 10^3^ tumor cells were injected into BALB/C nude mice, respectively. Each value is the mean tumor volume + SE. *P < 0.05. (**G**) Tumor incidence and estimated frequency of cancer stem cells. The number indicates tumor-initiation incidence in BALB/C nude mice. Cancer stem cell frequency was calculated by Extreme Limiting Dilution Analysis (ELDA) software. CI *=* confidence interval.

To address the functions of BORIS in cervical CSCs/CICs, we performed BORIS gene knockdown experiments using BORIS gene-specific siRNAs. Three different BORIS-specific siRNAs (siRNA #1, #2 and #3) could suppress the expression of BORIS by more than 90% in CaSki cells (Figure 3A). BORIS gene knockdown significantly inhibited sphere formation of CaSki cells, indicating that BORIS has a role in the maintenance of CSCs/CICs (Figure 3B and 3C). However, spheres smaller than 100 μm were observed for siRNA #3-transfected CaSki cells (Figure 3C).

A recent study showed that BORIS has 23 distinct mRNA variants generated from alternative splicing and that they encode 17 different BORIS peptides. These variants are classified into six distinct subfamilies (subfamily (sf) 1 through sf6) based on common 3′ sequences [30]. We thus designed BORIS sf-specific primer pairs and performed RT-PCR using sphere-culture and serum-culture CaSki and MS751 cells and testis tissue to determine which sf is expressed in CSCs/CICs (Figure 3D). BORIS sf 1, 2, 3 and 4 were detected in serum-culture CaSki cells, and BORIS sf 1, 2, 4 and 6 were detected in sphere-culture CaSki cells. BORIS sf4 was detected in serum-culture MS751 cells, and BORIS sf1, 3 and 6 were detected in sphere-culture MS751 cells. The BORIS expression pattern was different from that in the testis. As previously reported, BORIS variants are expressed in a different manner in each cell line [30, 31]. Our data indicated that sf1 and sf4 are expressed in both CSCs/CICs and non-CSCs/CICs and that BORIS sf6 is specifically expressed in CSCs/CICs.

To determine which sf has a role in the maintenance of CSCs/CICs, we created several BORIS gene constructs including BORIS variants B0 (sf1), B3 (sf4), B6 (sf6) and C7 (sf6). Each construct was transfected into CaSki and TC-S cells, and overexpressed sublines were established. BORIS variant overexpression was confirmed by RT-PCR and qRT-PCR (Supplementary Figure S1). A sphere formation assay revealed that TC-S cells in which BORIS variant B6 (sf6) was overexpressed had significantly higher sphere-forming ability than the abilities of mock and other sf-transfected cells (Figure 3E). CaSki cells with overexpression of BORIS B0 (sf1), B6 (sf6) and C7 (sf6) showed higher sphere forming ability than that of mock-transfected cells (Figure 3E). Thus, BORIS B6 might commonly have a role in the maintenance of CSCs/CICs.

Tumor-initiating ability in immune-deficient nude mice was addressed by using Mock, BORIS B0, BORIS B3, BORIS B6 and BORIS C7-overexpressed CaSki cells. CaSki/BORIS B6 cells formed significantly larger tumors than tumors derived from other cells (Figure 3F). Furthermore, initiation of tumor formation was observed in 6 of 6 mice (injection of 10^3^ cells), 4 of 6 mice (injection of 10^2^ cells) and 0 of 6 mice (injection of 10 cells) in which xenotransplantation of CaSki/BORIS B6 cells was performed, whereas initiation of tumor formation was observed in only 1 of 6 mice (injection of 10^3^ cells) and 1 of 6 mice (injection of 10^2^ cell) and 0 of 6 mice (injection of 10 cells) in which xenotransplantation of CaSki/mock cells was performed (Figure 3G). Estimated cancer stem cell frequency of CaSki/BORIS B6 cells was 1 in 108 by ELDA [25], and this is the highest CSC/CIC frequency among subfamily transfectants (Figure 3G).

To confirm the BORIS sf6 in tumor samples, RT-PCR using total RNAs isolated from cervical cancer FFPE samples were performed. BORIS^high^ cases (Pt1 – Pt12) were used. BORIS sf6 transctipts were detectable in 8 of 12 cases (Figure 2G) indicating that BORIS sf6 transcrits were expressed in human cervical cancer tissues.

### BORIS sf6-targeting immunotherapy can recognize cervical CSCs/CICs

**Figure 4:**
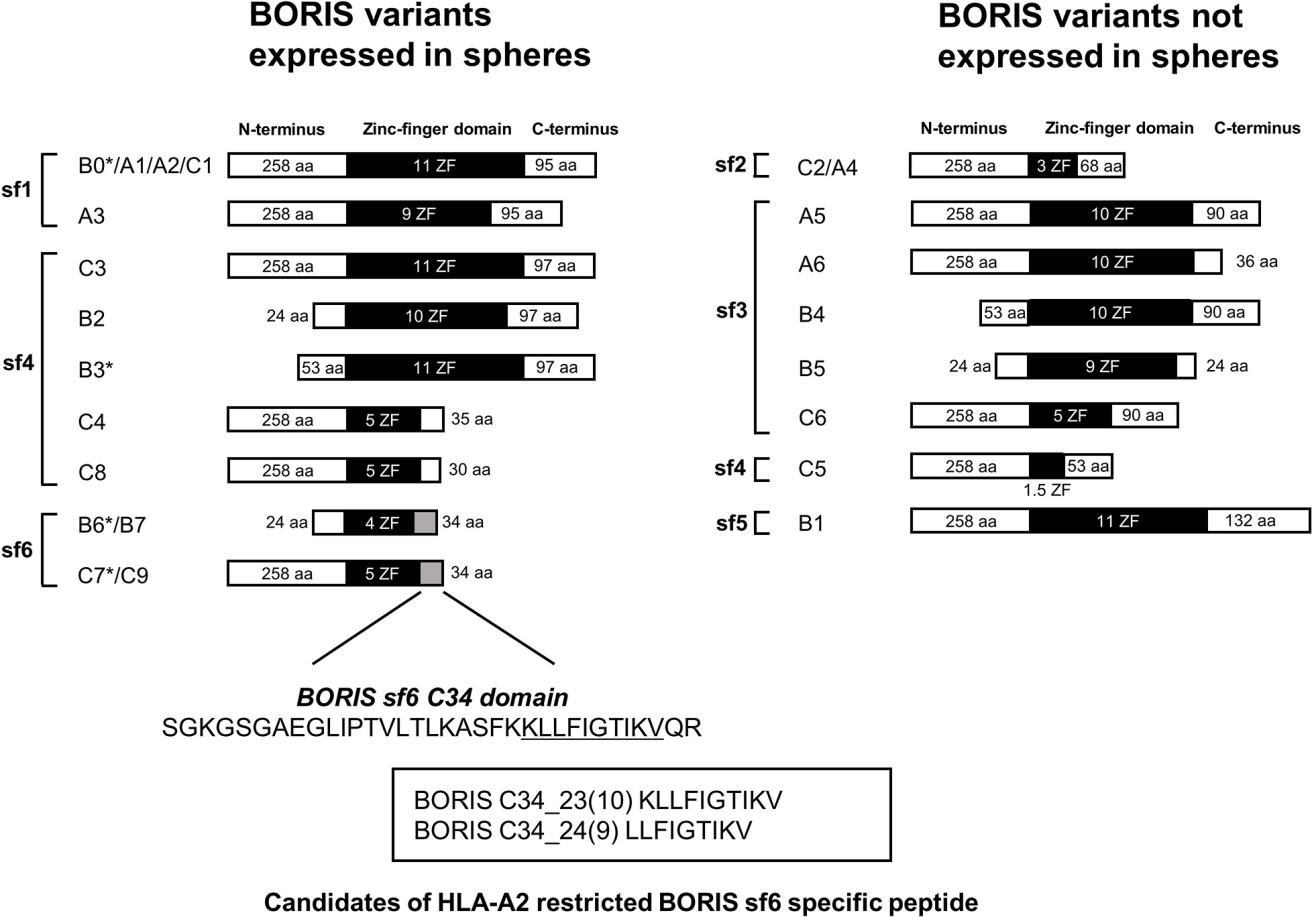
Schema of BORIS variants expressed in cercvical cancer stem cells BORIS variants were divided into two groups according to the expression in spheres derived from CaSki and MS751 cells. Asterisks indicate variants for which we examined the functions of by overexpression. We designed HLA-A2-restricted peptides from the C-terminus domain of BORIS sf6. Aa: amino acids Sf: subfamily ZF: zinc finger.

**Figure 5:**
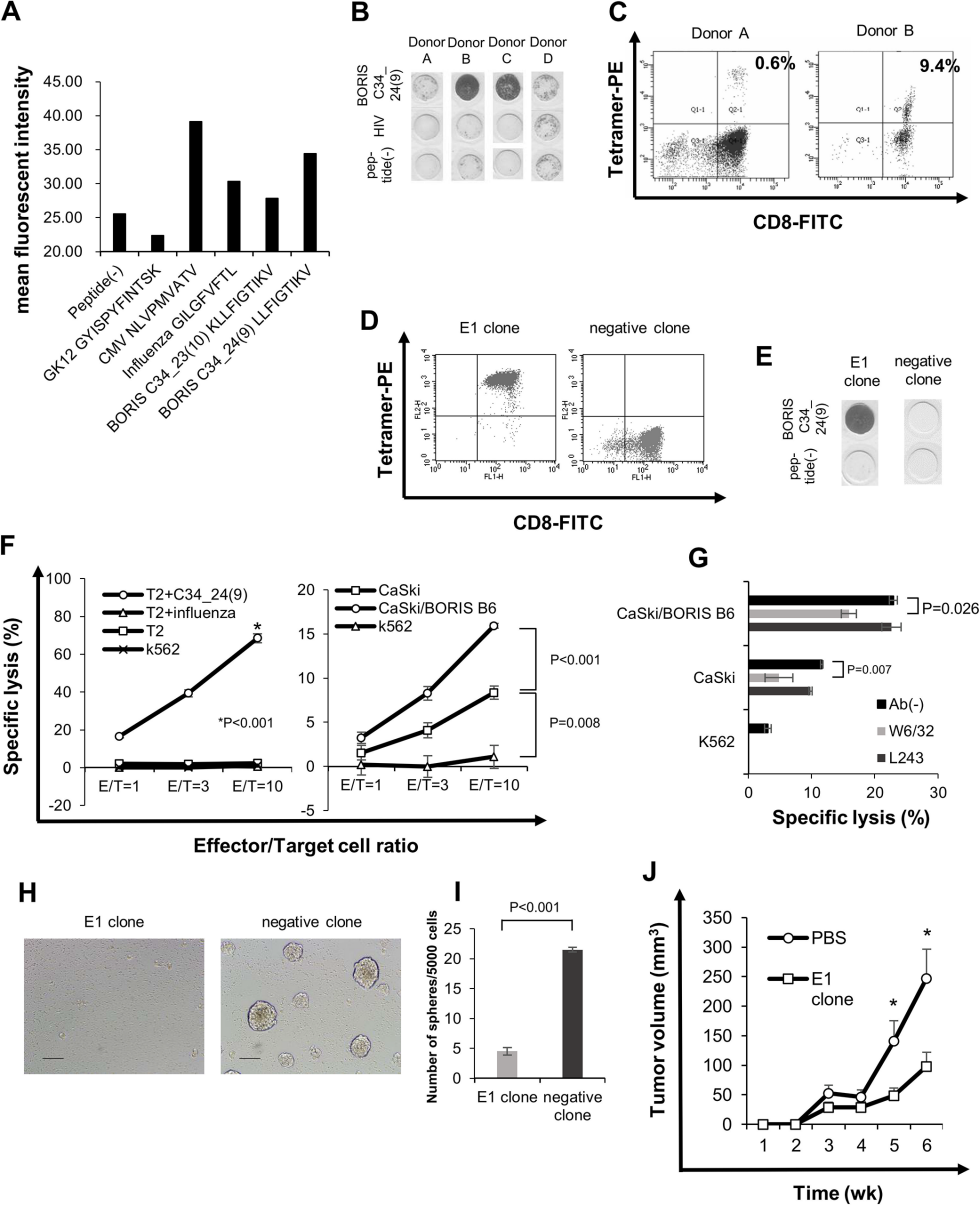
BORIS sf 6-specific CTL response can suppress sphere formation (**A**) Peptide binding assay. Binding affinity was evaluated by comparing mean fluorescence intensity of HLA-A2 expression in the presence of peptide pulsation to mean fluorescence intensity in the absence of the peptide. CMV and influenza peptides were used as positive controls, and GK12 peptide was used as a negative control. (**B**) ELISPOT assay. BORIS C34_24(9) peptide-specific cytotoxic T cell (CTL) induction was performed and assessed using the interferon (IFN)-γ enzyme-linked immunospot (ELISPOT) assay. HLA-A*0201-positive PBMCs were obtained from four healthy donors. Donors A, B and C were HLA-*A0201-positive and donor D was HLA-*A0206-positive. (**C**) Tetramer assay of BORIS C34_24(9)-specific CTLs. Fluorescence-activated cell sorting (FACS) was performed with PE-conjugated BORIS C34_24(9) peptide/HLA-A*0201 tetramer and anti-CD8-FITC antibody. BORIS C34_24(9) peptide/HLA-A*0201 tetramer-positive cells were directly sorted and a CTL clone was established. (**D**) Tetramer assay of a BORIS C34_24(9) peptide-specific CLT clone, E1. CTL clone E1 and negative CTL clone were stained by PE-conjugated BORIS C34_24(9) peptide/HLA-A*0201 tetramer and anti-CD8-FITC antibody and analyzed. (**E**) ELISPOT assay of CTL clone E1. BORIS C34_24(9) peptide specificity of CTL clone E1 and negative clone evaluated by the ELISPOT assay. (**F**) LDH release cytotoxicity assay. Specific cytotoxicity for peptide-pulsed T2 cells was aexamined (left panel). Influenza peptide-pulsed T2 cells, peptide (−) T2 cells and K562 cells were uses as negative controls. Specific cytotoxicity for CaSki and CaSki/BORIS B6 cells was examined (right panel). K562 cells were used as a negative control. Each value is the mean ± SE. (**G**) Blocking by anti-HLA-class I antibody. Cytotoxicity of the E1 clone for CaSki cells and CaSki/BORIS B6 cells was examined using anti-HLA class I mAb W6/32 and anti MHC-class II mAb L243. Each value is the mean ± SE. (H and I) Sphere formation in the presence of BORIS C34_24(9)-specific CTL clone. Five × 10^3^ CaSki cells and 5 × 10^4^ E1 CTL clone or CTL negative clone were co-cultured in a 96-well ultra low attachment plate. On co-culture day 8, a microscope photograph was taken (**H**). Original magnification is × 100. Size bar is 100 μm. The numbers of spheres were counted (**I**). Each value is the mean ± SE. (**J**) Tumor growth of CaSki cells in a therapeutic adoptive transfer model. Each value is the mean ± SE. Asterisks indicate statistically significant difference (*p* < 0.05).

Since BORIS sf6 (B6) has a role in the maintenance of cervical CSCs/CICs, BORIS B6 might be a suitable and reasonable target for cervical CSC/CIC-specific immunotherapy. Most of the amino acid sequence of BORIS B6 is shared with other BORIS variants; however, the C34 domain is unique for BORIS sf6 (B6) (Figure 4). We thus screened for potential antigenic peptides coded in the C34 domain, and we found two candidate antigenic peptides (BORIS C34_24(9) and BORIS C34_23(10)) that carry an HLA-A2-binding motif (Figure 4). A peptide binding assay revealed that BORIS C34_24(9) peptide has high binding affinity to HLA-A2, whereas BORIS C34_23(10) peptide has low binding affinity (Figure 5A). Thus, BORIS C34_24(9) peptide was used for further analysis.

To address the immunogenicity of BORIS C34_24(9) peptide, peptide-specific CTLs were induced using three HLA-A*0201^+^ healthy volunteer donors (donors A, B, and C) and one HLA-A*0206^+^ donor (donor D). PBMCs of four healthy volunteer donors were stimulated using BORIS C34_24(9) peptide and then the reactivity for the peptide was evaluated using an IFN-γ ELISpot assay and tetramer assay. BORIS C34_24(9)-specific interferon-γ spots were observed for CD8^+^ T cells from donors A, B and C but not for CD8^+^ T cells from donor D by the ELISPOT assay (Figure 5B). A PE-conjugated BORIS C34_24(9) peptide/HLA-A*0201 tetramer-positive population was observed for CD8^+^ T cells from donor A and donor B, and the positive rates were 0.6% and 9.4%, respectively (Figure 5C). PBMCs of donor C could not be analyzed by the tetramer assay due to the small number of cells.

To further analyze BORIS C34_24(9) peptide-specific CTLs, we established BORIS C34_24(9) peptide-specific CTL clones by sorting of tetramer-positive T cells and limiting dilution methods. We successfully generated 8 CTL clones specific for BORIS C34_24(9) peptide from donor A and 6 clones from donor B. BORIS C34_24(9) peptide-specificities of CTL clones were confirmed by the tetramer assay and ELIPOT assay (Figure 5D and 5E). BORIS C34_24(9) peptide-negative clones were obtained by only limiting dilution. The cell growth of CTL clone E1 was better than that of other clones, and we thus used the E1 clone for further analysis. An LDH release cytotoxicity assay revealed that the E1 clone showed specific cytotoxicity for BORIS C34_24(9) peptide-pulsed T2 cells compared with cytotoxicity for control peptide-pulsed T2 cells, T2 cells and K562 cells (Figure 5F). To determine whether the BORIS C34_24(9)-specific CTL clone can recognize endogenously presented BORIS C34_24(9) peptide of BORIS sf6-positive cancer cells, we performed an LDH release assay using BORIS B6/CaSki and CaSki cells. The E1 clone showed significantly higher cytotoxicity for CaSki cells than for K562 negative control cells. The E1 clone also showed significantly higher cytotoxicity for CaSki/BORIS B6 cells than for CaSki cells (Figure 5F). The cytotoxicities for CaSki cells and CaSki/BORIS B6 cells were significantly inhibited by HLA class I antibody (W6/32) (Figure 5G). The results indicate that BORIS C34_24(9) peptide is an immunogenic epitope and that the endogenously processed peptide is presented on the surface of CaSki cells.

Finally, we investigated whether BORIS C34_24(9)-specific CTLs suppress the sphere forming ability of cervical CSCs/CICs. Five thousand CaSki cells were co-cultured with 5 × 10^4^ cells of the E1 clone or negative clone for 7 days in a 96-well ultra low attachment plate. The E1 clone significantly suppressed sphere formation of CaSki cells compared with the negative clone (Figure 5H and 5I). Furthermore, CTL clone E1 significantly suppressed the tumor growth *in vivo* (Figure 5J). The results indicate that the BORIS C34_24(9) peptide-specific CTL clone can recognize cervical CSCs/CICs.

## DISCUSSION

In this study, we isolated cervical CSCs/CICs by sphere culture. Previous studies showed that cervical CSCs/CICs can be isolated by sphere culture and that ALDH^high^ cells can be isolated by the ALDEFLUOR assay [12, 13]. The cells forming spheres showed a dormant cell cycle status, resistance to radiothepray and chemotherapy, and higher expression levels of stem cell-related genes. These results indicate that spheres and ALDH^high^ cells are proper sources of cervical CSCs/CICs.

We identified BORIS as a cervical CSC/CIC-related gene. BORIS shares an 11 zinc-finger domain with its paralogue CTCF, and it has a role as an epigenetic factor including chromatin remodeling via histone modifications [32–34] and germline imprinted gene methylation [35]. CTCF is an insulator and tumor suppressor gene, whereas BORIS exhibits oncogenetic properties including an anti-apoptotic ability via upregulation of the human Telomerase Reverse Transcriptase (hTERT) gene in embryonic and ovarian tumor cells [36]. High expression level of BORIS determined by immunohistochemistry and high BORIS/CTCF mRNA ratio are related to poorer prognosis for patients with esophageal cancer and epithelial ovarian cancer, respectively [37, 38]. In this study, we showed that a high BORIS protein expression level determined by immunohistochemistry is related to poorer prognosis for patients with FIGO stage III/IV cervical cancer. These observations indicate that a high level of BORIS expression might be a novel prognostic marker for cancers. Alberti *et al.* isolated 3–5% of BORIS-positive cells from an embryonic cancer cell line using a BORIS mRNA-targeting molecular beacon. BORIS-positive cells expressed cancer stem genes including CD44, ALDH1, NANOG, OCT4 and SOX2 [39]. We previously reported that BORIS is preferentially expressed in CSCs/CICs isolated as SP cells of colon cancer [29]. We confirmed BORIS expression in cervical CSCs/CICs by qRT-PCR in the present study. Monk *et al.* showed that BORIS is also expressed in embryonic stem (ES) cells and that it is co-localized with OCT4 and NANOG [40]. These observations indicate that BORIS is related to CSCs/CICs and might associate with stem cell-related transcription factors including OCT4 and NANOG.

In this study, we performed RT-PCR to investigate the expression pattern of BORIS classified by 6 subfamilies in CSCs/CICs. Original BORIS (B0) has an 11 zinc-finger domain, N258 domain and C95 domain. BORIS isoproteins have various numbers of zinc finger domains and a distinct N-terminus domain and c-terminus domain. Among the BORIS subfamilies, BORIS sf6, which has only 4 or 5 zinc-finger domains and a distinct C34 domain, is more specifically expressed in CSCs/CICs. Moreover, the sphere formation assay and tumor xenograft model in BALB/C nude mice revealed that BORIS variant B6 (sf6) is involved in the maintenance and high tumor initiating ability of CSCs/CICs. The function of each BORIS variant is still elusive; however, Pugacheva *et al.* reported that DNA binding efficiency of BORIS isoproteins depends on the number of zinc-finger domains. They demonstrated by using an electrophoresis mobility shift assay (EMSA) that H19 imprinting control region (ICR), which is a common binding resion of CTCF and BORIS, was required to bind BORIS isoproteins with at least nine zinc fingers, while only five zinc fingers were required for binding the promoter of the mouse cerebroside sulfotransferase (CST) gene. Furthermore, they showed that the N258 amino terminus of BORIS isoproteins in common is required to activate the CST promoter [30]. Their findings and our findings indicate that each BORIS isoprotein has a distinct gene regulatory network modulating different gene transcription or competing with another variant or CTCF and that BORIS variant B6 might be specifically related to gene expression of cancer stemness.

BORIS is promising as an immunotherapeutic target because of its exclusive expression pattern in normal organs and its oncogenetic properties. Aberrant expression of BORIS has been reported in various primary cancers including glioblasma [41], melanomas [42], head and neck squamous cell carcinoma [43], esophageal cancer [37], breast cancer [44, 45], prostate cancer [46], uterine corpus cancer [47, 48] and ovarian cancer [49] at mRNA or protein levels. Thus, immunotherapy targeting BORIS might be beneficial for diverse cancer patients. The potency of BORIS for immunotherapy was adverted with a mouse model in previous studies. Previous studies showed that a plasmid encoding a mutated murine BORIS (pmBORIS) DNA vaccine could induce murine CD8^+^ and CD4^+^ T lymphocyte responses and exhibited an anti-cancer effect [50, 51]. However, there is no report in which the T cell immune response for human BORIS is described. In this report, we describe the identification of a CTL epitope encoded by BORIS protein for the first time. BORIS C34_24(9) peptide is coded in the C34 domain of CSC/CIC-specific BORIS variant sf6. BORIS C34_24(9) peptide-specific CTL clone E1 recognized BORIS B6-overexpressing cells, indicating that BORIS C34_24(9) peptide is endogenously expressed and presented by HLA-A2. Importantly, the CTL clone E1 suppressed the sphere formation ability of CaSki cells, indicating that BORIS C34_24(9)-specific CTLs can kill CBDCA and radiotherapy-resistant sphere cells. Therefore, cancer immunotherapy using BORIS C34_24(9) peptide might be a promising approach to treat chemotherapy and radiotherapy-resistant cervical cancers.

In conclusion, we demonstrated that cervical cancer spheres showed characteristics of CSCs/CICs and that BORIS sf6 is involved in cervical cancer stemness. BORIS C34_24(9) peptide encoded by BORIS sf6 is a candidate antigenic peptide for cervical CSC/CIC-targeting immunotherapy.

## MATERIALS AND METHODS

### Ethics statement

Mice were maintained and experimented on in accordance with the guidelines of and after approval by the Committee of Sapporo Medical University School of Medicine, Animal Experimentation Center under permit number 08–006. Any animal found unhealthy or sick was promptly euthanized. All studies were approved by the Institutional Review Board (IRB) of Sapporo Medical University Hospital. Written informed consent was obtained from all patients and healthy blood donors according to the guidelines of the Declaration of Helsinki.

### Cell lines

Human cervical squamous cell carcinoma (SCC) cell lines CaSki, MS751 and ME180, human lymphoblastoid cell line T2, transporter associated with antigen processing (TAP)-deficient, and erythroleukemia cell line K562 were purchased from American Type Culture Collection (ATCC, Manassas, VA, USA). SKG3b cells were purchased from Japanese Collection of Research Bioresources Cell Bank (JCRB Cell Bank, Osaka, Japan). TC-S cells were a kind gift from Dr. Kawabata (Department of Obstetrics and Gynecology, Faculty of Medicine, Toyama Medical and Pharmaceutical University, Toyama, Japan) [21]. PLAT-A cells were a kind gift from Dr. T. Kitamura (The University of Tokyo, Tokyo, Japan) [22]. CaSki, T2 and K562 cells were cultured in RPMI-1640 (Sigma-Aldrich, St Louis, MO, USA). MS751 and TC-S cells were cultured in Minimum Essential Medium (MEM; Life Technologies, Carlsbad, CA, USA). ME180 cells were cultured in McCoy's 5a Medium (Life Technologies). SKG3b cells were cultured in Ham's F12 Medium (Life Technologies). To all media, 10% fetal bovine serum (FBS) was added. PLAT-A cells was cultured in Dulbecco's Modified Eagle's Medium (DMEM; Life Technologies) supplemented with 10 mg/mL blasticidin and 1 mg/mL puromycin. Cells were incubated in a humidified 5% CO_2_ incubator at 37°C.

### Sphere culture

Sphere culture was performed as described previously. [23] Briefly, 10^3^ cells were plated, and the number of spheres in an area of 100 μm in diameter was counted on day 14. The sphere culture medium was serum-free DMEM/F12 (Life Technologies) supplemented with N-2 supplement (Wako, Osaka, Japan), 20 mg/ml recombinant human epithelial growth factor, 10 mg/ml human basic fibroblast growth factor (R & D Systems), 4 μg/ml heparin (AY pharma, Tokyo, JAPAN) and 1% penicillin and streptomycin.

### Radioresistance and chemoresistance assay

The effects of radiation and a chemotherapy agent on cell viability of spheres and serum-cultured cells were evaluated with the WST-1 assay. One thousand CaSki and TC-S cells were plated in a 96-well plate with 100 μl serum culture medium as serum-cultured cells, and ten thousand of CaSki and TC-S cells were plated in an ultra-low attachment 96-well plate (Corning) with sphere culture medium as sphere cultured-cells. After 72 hours of preculture, cells were treated by irradiation (5 Gy or 10 Gy) or by chemotherapy (10, 50, 100 and 500 μg/ml of CBDCA). Seventy-two hours after treatments, relative cell viability was analyzed using the WST-1 Cell Proliferation Assay System (Wako, Osaka, Japan) according to the instructions of the manufacturer.

### Cell cycle assay

Adherent cultured cells were enzymatically dissociated by incubation in a trypsin-EDTA solution at 37°C, and spheres were mechanically dissociated by pipetting. Cells were fixed with 70% ethanol and resuspended in PBS containing 250 μg/ml RNase A (Sigma-Aldrich) for 30 minutes at 37°C, followed by staining with 50 μg/ml propidium iodide for 10 minutes at 4°C in the dark. Stained cells were filtered into a conical tube with a 35 μm nylon filter and analyzed with a FACSCalibur (BD Biosciences, San Jose, CA, USA) and Mod-Fit cell cycle analysis program.

### Immunohistochemistry (IHC)

Immunohistochemical staining of BORIS was performed as described previously [24] using formalin-fixed, paraffin-embedded sections of biopsy specimens from 38 patients with International Federation of Gynecology and Obstetrics (FIGO) stage III or IV of untreated cervical squamous cell carcinoma. All patients received initial treatment at Sapporo Medical University Hospital during the period from 2001 to 2011. A rabbit anti-BORIS polyclonal antibody (HPA001472, Sigma-Aldrich) was used at a 1:100 dilution. For evaluation of BORIS staining, the cases were divided into two groups (BORIS^high^ group and BORIS^low^ group) by a cut line of 50% of positive rates.

### Total RNA isolation and microarray preparation

Total RNA was isolated from collected cells using an RNeasy Mini Kit (QIAGEN, Valencia, CA) following the manufacturer's protocol. We used the commercially available Low Input Quick Amp Labeling Kit (Agilent Technologies). Purified total RNA (3 μg) was reverse-transcribed to generate double-stranded cDNA using an oligo dT T7 promoter primer and reverse transcriptase. Then cRNA was synthesized using T7 RNA polymerase, which simultaneously incorporated Cy3- or Cy5-labeled cytidine triphosphate. During this process, the samples of sphere cells were labeled with Cy5, whereas the adherent cultured cells were labeled with Cy3 as control cells. The quality of cRNA was again checked using Nano Drop. Cy3-labeled cRNA and Cy5-labeled cRNA were combined and then fragmented in a gene expression hybridization kit (Agilent Technologies). Then the labeled cRNAs were hybridized to a 60-mer probe oligonucleotide microarray (G4845A human GE 4x44K V2 Microarray kit) and incubated for 20 hours at 50°C. The fluorescent intensities were determined by an Agilent Technologies Scanner G2505C. Samples of sphere cells were labeled with Cy3, whereas adherent cultured cells were labeled with Cy5. Microarray raw data and processed data have been deposited in the NCBI GEO database (GSE65499).

Total RNA from formalin-fixed, paraffin-embedded (FFPE) samples were isolated using NucleoSpin totalRNA FFPE kit according to manufactures protocol (TaKaRa Bio., Kusatsu, JAPAN). BORIS IHC positive cases (Pt1–Pt12) were used.

### Reverse transcription PCR (RT-PCR) and quantitative RT-PCR (qRT-PCR)

RT-PCR and qRT-PCR were performed as described previously [23]. Human normal tissue panel (Human MTC panel I and Human MTC panel II, TaKaRa Bio. Inc., Kusatsu, Japan) were used. The primer sequences used for cDNA amplification are summerized in Table S1. Quantitative PCR was performed using a Stepone Real-Time PCR System (Applied Biosystems, Foster City, CA, USA) according to the manufacturer's protocol. *SOX2* (Hs01053049_s1), *POU5F1* (Hs00999632_g1), *NANOG* (Hs04260366_g1), *KLF4*(Hs00358836_m1), and *CTCFL*(Hs00966548_g1, which is specific for CTCFL(BORIS) variants including B0, B3, B6 and C7, primers and probes were designed by the manufacturer (TaqMan Gene expression assays; Applied Biosystems). Each experiment was performed in triplicate. Expression of each gene was normalized to that of GAPDH mRNA. Fold change was determined by the ΔΔCt method.

### siRNA-mediated knockdown

BORIS siRNAs (SR315374; OriGene Technologies, Rockville, MD, USA) were transfected using Lipofectamine RNAiMAX reagent (Invitrogen) according to the protocol of the manufacturer. Cells were transfected with siRNA 48 hours before analysis. Universal scrambled siRNA (SR30004; Origene Technologies) was used as a negative control. BORIS knockdown was confirmed by qRT-PCR.

### Retroviral gene transduction and generation of stable transformants

Transduction of genes into cells was carried out using a retrovirus-mediated method as described previously [22]. cDNAs coding BORIS variants B0, B3, B6, C7 were cloned into the pMXs-puro retroviral vector (kind gift from Dr. T. Kitamura, Tokyo).

### Mouse xenograft assay and CTL adoptive transfer

CaSki-transduced BORIS variants (CaSki/mock, CaSki/BORIS B0, CaSki/BORIS B3, CaSki/BORIS B6 and CaSki/BORIS C7) were suspended at 10^2^, 10^3^ and 10^4^ cells in 100 μl PBS mixed with Matrigel (BD) at a 1:1 volume and injected subcutaneously into the backs of 4-week-old female BALB/C nude mice. Tumor size was assessed weekly using a caliper and calculated using the following formula: tumor size (mm^3^) *=* (longest diameter × shortest diameter^2^)/2. Cancer stem cell frequency was calculated by web program Extreme Limiting Dilution Analysis (ELDA; http://bioinf.wehi.edu.au/software/elda/) software [25].

For CTL adoptive transfer, 10^4^ of CasKi cells were injected NOD/SCID mice. On day 21 and day 28, 10^7^ of CTL clone E1 were suspended in 100 ul of PBS were injected intravenously. PBS was used as a negative control. The tumor growth were assed weekly.

### Synthetic peptides and peptide binding assay

Two peptides, BORIS C34_23(10) (KLLFIGTIKV) and BORIS C34_24(9) (LLFIGTIKV), were designed designed by BIMAS web site (http://www-bimas.cit.nih.gov/molbio/hla_bind/). Peptide binding affinity to HLA-A2 was assessed using T2 cells as described previously. [26] A cytomegalovirus (CMV) peptide (NLVPMVATV; HLA-A*0201) and an influenza viral peptide (GILGFVFTL; HLA-A*0201) were used as positive controls, and GK12 (GYISPYFINTSK) was used as a negative control.

### Cytotoxic T lymphocyte (CTL) induction and interferon-γ ELISPOT assay

CTL induction was performed as described previously. [27] HLA-A2-positive PBMCs were isolated from four healthy volunteer donors (Donors A, B and C are HLA-A*0201-positive and donor D is HLA-A*0206-positive.). An IFN-γ ELISPOT assay was performed as described previously. [28] Five × 10^4^ CTLs were incubated with 5 × 10^4^ T2 cells per well pulsed with each peptide (20 μg/mL). After incubation overnight at 37°C, IFN-γ spots were developed and assessed.

### Peptide-MHC tetramer assay and establishment of CTL clone

BORIS C34_24(9) peptide/HLA-A2 tetramers were synthesized by MBL (Nagoya, Aichi, JAPAN). CTLs were stained by the tetramer at 4°C for 30 min at a 1:20 dilution and then stained for a further 20 min with fluorescein isothiocyanate (FITC)-conjugated mouse anti-CD8 monoclonal antibody (1:20 dilution). Stained CTLs were analyzed with a FACSaria II (BD Biosciences) or a FACScalibur. To obtain CTL clones, tetramer-positive single cell sorting was performed with FACSaria II, and standard limiting dilution was done as described previously [28].

### LDH release cytotoxicity assay

The antigen-specific lytic activity of CTL clones was evaluated using an LDH Cytotoxicity Detection Kit (Takara Bio, Otsu, Japan) following the manufacturer's protocol. Target cells (10000 cells/well) were incubated with various numbers of effector cells for 7 hrs at 37°C in 96-well V-bottomed plates, and cytotoxicity was calculated using the culture supernatants. The percentage of specific lysis was calculated as [(experimental release − spontaneous release)/(maximum release − spontaneous release)] × 100. Cytotoxicity blocking assays were performed using monoclonal antibodies. Tumor targets were incubated with hybridoma culture supernatants of anti-HLA-class I antibody (W6/32) or anti-HLA-DR antibody (L243) for 1 hr at room temperature.

### Statistical analysis

Data are presented as means ± SE or SD. Fisher's exact test or Student's *t*-test was used to determine the significance of associations between characteristic valuables. Differences in variables were assessed using Student's *t* test. Survival curves were constructed according to the Kaplan–Meier method, and differences between groups were tested by the log-rank test. Statistical significance was determined by the log-rank test. *P* < 0.05 was considered significant. Statistical analysis was done with GraphPad Prism (version 4.0 for Windows; GrapgPad Software Inc).

## SUPPLEMENTARY MATERIALS FIGURE AND TABLE


