# Supplementary material for: Brother of the regulator of the imprinted site (BORIS) variant subfamily 6 is involved in cervical cancer stemness and can be a target of immunotherapy
Source: Oncotarget. 2016 Feb 3;7(10):11223–37. doi: 10.18632/oncotarget.7165 (PMC4905468; doi:10.18632/oncotarget.7165)
Supplement: Supplementary file 1 [file oncotarget-07-11223-s001.pdf]

# Brother of the regulator of the imprinted site (BORIS) variant subfamily 6 is involved in cervical cancer stemness and can be a target of immunotherapy

## Supplementary Materials

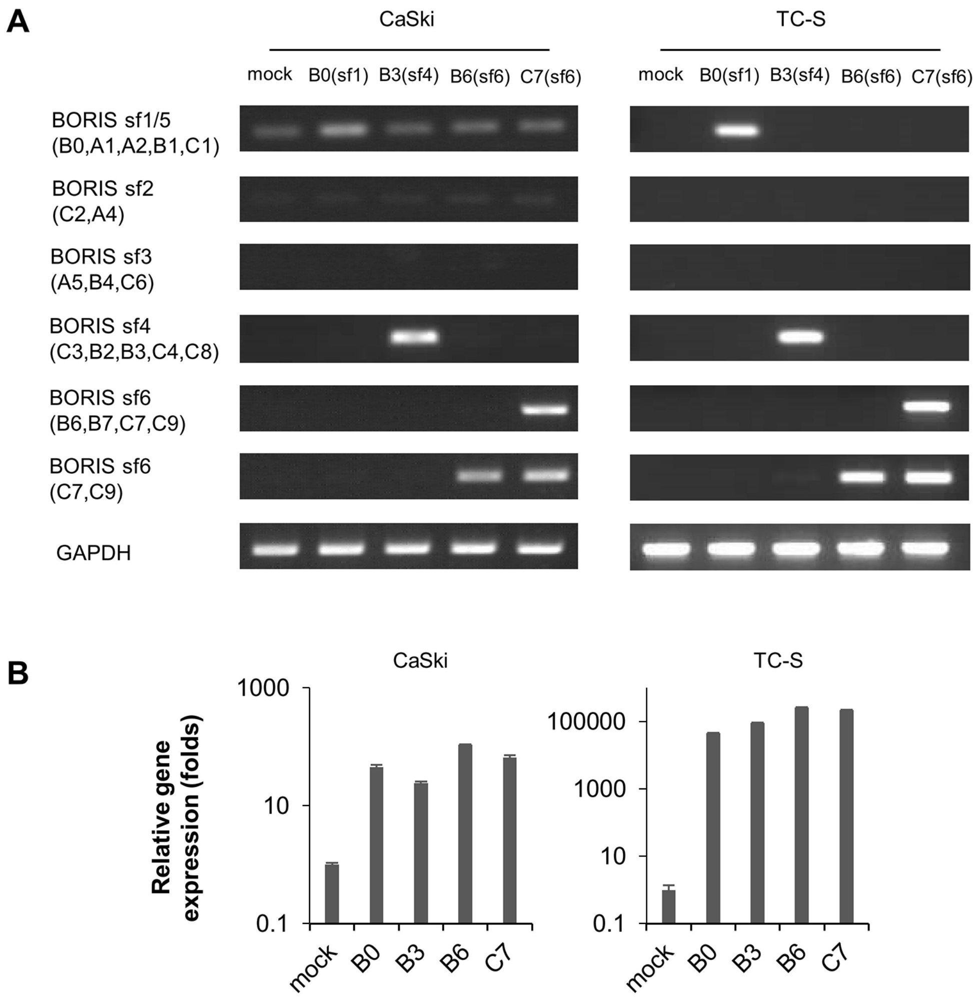


**Supplementary Figure S1: BORIS variant BO, B3, B6, C7 overexpression experiments.** (**A**) RT-PCR using specific primers for BORIS subfamilies in mock, BORIS BO, BORIS B3, BORIS B6 and BORIS C7-overexpressed CaSki cells. (**B**) QRT-PCR of BORIS. Data are shown as a comparison with the expression level in mock, BORIS BO, BORIS B3, BORIS B6 and BORIS C7-overexpressed CaSki cells. Data represent the means ± SD of RQ.

## Supplementary Table: S1 Summary of the gene expression in CasKi sphere-culture cells

| GenBank_accession | Description | Relative mRNA expression (fold over control) |
|---|---|---|
| NM_000311 | Homo sapiens prion protein (PRNP), transcript variant 1, mRNA [NM_000311] | 283.64 |
| NM_001062 | Homo sapiens transcobalamin I (vitamin B12 binding protein, R binder family) (TCN1), mRNA [NM_001062] | 154.58 |
| NM_002964 | Homo sapiens S100 calcium binding protein A8 (S100A8), mRNA [NM_002964] | 139.20 |
| NM_002421 | Homo sapiens matrix metallopeptidase 1 (interstitial collagenase) (MMP1), transcript variant 1, mRNA [NM_002421] | 118.10 |
| NM_002425 | Homo sapiens matrix metallopeptidase 10 (stromelysin 2) (MMP10), mRNA [NM_002425] | 92.71 |
| DQ080207 | gb\|Homo sapiens MIG7 (MIG7) mRNA, complete cds. [DQ080207] | 90.63 |
| NM_007350 | Homo sapiens pleckstrin homology-like domain, family A, member 1 (PHLDA1), mRNA [NM_007350] | 66.00 |
| NM_001657 | Homo sapiens amphiregulin (AREG), mRNA [NM_001657] | 62.12 |
| NM_001946 | Homo sapiens dual specificity phosphatase 6 (DUSP6), transcript variant 1, mRNA [NM_001946] | 55.39 |
| NM_003234 | Homo sapiens transferrin receptor (p90, CD71) (TFRC), mRNA [NM_003234] | 55.10 |
| NM_001712 | Homo sapiens carcinoembryonic antigen-related cell adhesion molecule 1 (biliary glycoprotein) (CEACAM1), transcript variant 1, mRNA [NM_001712] | 52.08 |
| NM_003483 | Homo sapiens high mobility group AT-hook 2 (HMGA2), transcript variant 1, mRNA [NM_003483] | 50.29 |
| NM_013409 | Homo sapiens follistatin (FST), transcript variant FST344, mRNA [NM_013409] | 49.02 |
| NM_021101 | Homo sapiens claudin 1 (CLDN1), mRNA [NM_021101] | 48.73 |
| NM_015444 | Homo sapiens transmembrane protein 158 (gene/pseudogene) (TMEM158), mRNA [NM_015444] | 45.77 |
| NM_005555 | Homo sapiens keratin 6B (KRT6B), mRNA [NM_005555] | 45.18 |
| NM_016040 | Homo sapiens transmembrane emp24 protein transport domain containing 5 (TMED5), transcript variant 1, mRNA [NM_016040] | 44.96 |
| NM_006919 | Homo sapiens serpin peptidase inhibitor, clade B (ovalbumin), member 3 (SERPINB3), mRNA [NM_006919] | 44.61 |
| NM_052886 | Homo sapiens mal, T-cell differentiation protein 2 (MAL2), mRNA [NM_052886] | 44.23 |
| NM_000576 | Homo sapiens interleukin 1, beta (IL1B), mRNA [NM_000576] | 42.81 |
| NM_004869 | Homo sapiens vacuolar protein sorting 4 homolog B (S. cerevisiae) (VPS4B), mRNA [NM_004869] | 39.95 |
| NM_012328 | Homo sapiens DnaJ (Hsp40) homolog, subfamily B, member 9 (DNAJB9), mRNA [NM_012328] | 38.76 |
| NM_000274 | Homo sapiens ornithine aminotransferase (OAT), nuclear gene encoding mitochondrial protein, transcript variant 1, mRNA [NM_000274] | 37.95 |
| NM_053056 | Homo sapiens cyclin D1 (CCND1), mRNA [NM_053056] | 37.56 |

| | | |
|---|---|---|
| NM_017526 | Homo sapiens leptin receptor overlapping transcript (LEPROT), transcript variant 1, mRNA [NM_017526] | 37.42 |
| NM_000210 | Homo sapiens integrin, alpha 6 (ITGA6), transcript variant 2, mRNA [NM_000210] | 37.00 |
| NM_005494 | Homo sapiens DnaJ (Hsp40) homolog, subfamily B, member 6 (DNAJB6), transcript variant 2, mRNA [NM_005494] | 36.79 |
| NM_017414 | Homo sapiens ubiquitin specific peptidase 18 (USP18), mRNA [NM_017414] | 36.78 |
| NM_002822 | Homo sapiens twinfilin, actin-binding protein, homolog 1 (Drosophila) (TWF1), mRNA [NM_002822] | 35.90 |
| NM_001779 | Homo sapiens CD58 molecule (CD58), transcript variant 1, mRNA [NM_001779] | 33.97 |
| NM_001423 | Homo sapiens epithelial membrane protein 1 (EMP1), mRNA [NM_001423] | 33.88 |
| NM_003064 | Homo sapiens secretory leukocyte peptidase inhibitor (SLPI), mRNA [NM_003064] | 32.16 |
| NM_005054 | Homo sapiens RANBP2-like and GRIP domain containing 5 (RGPD5), transcript variant 1, mRNA [NM_005054] | 30.64 |
| NM_014888 | Homo sapiens family with sequence similarity 3, member C (FAM3C), transcript variant 1, mRNA [NM_014888] | 30.09 |
| NM_002089 | Homo sapiens chemokine (C-X-C motif) ligand 2 (CXCL2), mRNA [NM_002089] | 29.87 |
| NM_001547 | Homo sapiens interferon-induced protein with tetratricopeptide repeats 2 (IFIT2), mRNA [NM_001547] | 29.83 |
| NM_002755 | Homo sapiens mitogen-activated protein kinase kinase 1 (MAP2K1), mRNA [NM_002755] | 29.78 |
| NM_080657 | Homo sapiens radical S-adenosyl methionine domain containing 2 (RSAD2), mRNA [NM_080657] | 29.34 |
| NM_002483 | Homo sapiens carcinoembryonic antigen-related cell adhesion molecule 6 (non-specific cross reacting antigen) (CEACAM6), mRNA [NM_002483] | 29.33 |
| NM_019597 | Homo sapiens heterogeneous nuclear ribonucleoprotein H2 (H') (HNRNPH2), transcript variant 1, mRNA [NM_019597] | 29.19 |
| NM_000600 | Homo sapiens interleukin 6 (interferon, beta 2) (IL6), mRNA [NM_000600] | 28.69 |
| NM_004661 | Homo sapiens cell division cycle 23 homolog (S. cerevisiae) (CDC23), mRNA [NM_004661] | 27.30 |
| NM_002353 | Homo sapiens tumor-associated calcium signal transducer 2 (TACSTD2), mRNA [NM_002353] | 27.17 |
| NM_015339 | Homo sapiens activity-dependent neuroprotector homeobox (ADNP), transcript variant 1, mRNA [NM_015339] | 26.51 |
| NM_014314 | Homo sapiens DEAD (Asp-Glu-Ala-Asp) box polypeptide 58 (DDX58), mRNA [NM_014314] | 26.48 |
| NM_002983 | Homo sapiens chemokine (C-C motif) ligand 3 (CCL3), mRNA [NM_002983] | 26.47 |
| NM_015137 | Homo sapiens EFR3 homolog A (S. cerevisiae) (EFR3A), mRNA [NM_015137] | 26.43 |

| | | |
|---|---|---|
| NM_001257 | Homo sapiens cadherin 13, H-cadherin (heart) (CDH13), mRNA [NM_001257] | 26.01 |
| NM_000043 | Homo sapiens Fas (TNF receptor superfamily, member 6) (FAS), transcript variant 1, mRNA [NM_000043] | 25.64 |
| NM_206831 | Homo sapiens DPH3, KTI11 homolog (S. cerevisiae) (DPH3), transcript variant 1, mRNA [NM_206831] | 25.11 |
| NM_021204 | Homo sapiens enolase-phosphatase 1 (ENOPH1), mRNA [NM_021204] | 25.07 |
| NM_003733 | Homo sapiens 2′-5′-oligoadenylate synthetase-like (OASL), transcript variant 1, mRNA [NM_003733] | 24.65 |
| NM_001017915 | Homo sapiens inositol polyphosphate-5-phosphatase, 145kDa (INPP5D), transcript variant 1, mRNA [NM_001017915] | 24.55 |
| NM_001005340 | Homo sapiens glycoprotein (transmembrane) nmb (GPNMB), transcript variant 1, mRNA [NM_001005340] | 24.45 |
| NM_002970 | Homo sapiens spermidine/spermine N1-acetyltransferase 1 (SAT1), transcript variant 1, mRNA [NM_002970] | 24.13 |
| NM_002717 | Homo sapiens protein phosphatase 2, regulatory subunit B, alpha (PPP2R2A), transcript variant 1, mRNA [NM_002717] | 24.07 |
| NM_014044 | Homo sapiens unc-50 homolog (C. elegans) (UNC50), mRNA [NM_014044] | 24.03 |
| NM_205842 | Homo sapiens NCK-associated protein 1 (NCKAP1), transcript variant 2, mRNA [NM_205842] | 24.00 |
| NM_016584 | Homo sapiens interleukin 23, alpha subunit p19 (IL23A), mRNA [NM_016584] | 23.79 |
| NM_031458 | Homo sapiens poly (ADP-ribose) polymerase family, member 9 (PARP9), transcript variant 1, mRNA [NM_031458] | 23.35 |
| NM_020672 | Homo sapiens S100 calcium binding protein A14 (S100A14), mRNA [NM_020672] | 23.06 |
| NM_152756 | Homo sapiens RPTOR independent companion of MTOR, complex 2 (RICTOR), mRNA [NM_152756] | 22.97 |
| NM_002468 | Homo sapiens myeloid differentiation primary response gene (88) (MYD88), transcript variant 2, mRNA [NM_002468] | 22.86 |
| NM_006007 | Homo sapiens zinc finger, AN1-type domain 5 (ZFAND5), transcript variant c, mRNA [NM_006007] | 22.74 |
| NM_005034 | Homo sapiens polymerase (RNA) II (DNA directed) polypeptide K, 7.0kDa (POLR2K), mRNA [NM_005034] | 22.65 |
| NM_001730 | Homo sapiens Kruppel-like factor 5 (intestinal) (KLF5), mRNA [NM_001730] | 22.60 |
| NM_182715 | Homo sapiens synaptophysin-like 1 (SYPL1), transcript variant 2, mRNA [NM_182715] | 22.51 |
| NM_003715 | Homo sapiens USO1 vesicle docking protein homolog (yeast) (USO1), mRNA [NM_003715] | 22.26 |
| NM_007214 | Homo sapiens SEC63 homolog (S. cerevisiae) (SEC63), mRNA [NM_007214] | 22.15 |
| NM_003122 | Homo sapiens serine peptidase inhibitor, Kazal type 1 (SPINK1), mRNA [NM_003122] | 21.92 |

| | | |
|---|---|---|
| NM_032842 | Homo sapiens transmembrane protein 209 (TMEM209), mRNA [NM_032842] | 21.61 |
| NM_005792 | Homo sapiens M-phase phosphoprotein 6 (MPHOSPH6), mRNA [NM_005792] | 21.53 |
| NM_006330 | Homo sapiens lysophospholipase I (LYPLA1), mRNA [NM_006330] | 21.31 |
| NM_144505 | Homo sapiens kallikrein-related peptidase 8 (KLK8), transcript variant 2, mRNA [NM_144505] | 21.15 |
| NM_002357 | Homo sapiens MAX dimerization protein 1 (MXD1), mRNA [NM_002357] | 21.05 |
| NM_004162 | Homo sapiens RAB5A, member RAS oncogene family (RAB5A), mRNA [NM_004162] | 20.93 |
| NM_012170 | Homo sapiens F-box protein 22 (FBXO22), transcript variant 2, mRNA [NM_012170] | 20.83 |
| NM_023938 | Homo sapiens chromosome 1 open reading frame 116 (C1orf116), transcript variant 1, mRNA [NM_023938] | 20.82 |
| NM_020665 | Homo sapiens transmembrane protein 27 (TMEM27), mRNA [NM_020665] | 20.75 |
| NM_198793 | Homo sapiens CD47 molecule (CD47), transcript variant 2, mRNA [NM_198793] | 20.70 |
| NM_001185156 | Homo sapiens interleukin 24 (IL24), transcript variant 3, mRNA [NM_001185156] | 20.64 |
| NM_005213 | Homo sapiens cystatin A (stefin A) (CSTA), mRNA [NM_005213] | 20.32 |
| NM_018139 | Homo sapiens chromosome 14 open reading frame 104 (C14orf104), transcript variant 1, mRNA [NM_018139] | 20.06 |
| NM_016045 | Homo sapiens slowmo homolog 2 (Drosophila) (SLMO2), mRNA [NM_016045] | 20.04 |
| NM_002814 | Homo sapiens proteasome (prosome, macropain) 26S subunit, non-ATPase, 10 (PSMD10), transcript variant 1, mRNA [NM_002814] | 19.96 |
| NM_014506 | Homo sapiens torsin family 1, member B (torsin B) (TOR1B), mRNA [NM_014506] | 19.82 |
| NM_178034 | Homo sapiens phospholipase A2, group IVD (cytosolic) (PLA2G4D), mRNA [NM_178034] | 19.76 |
| NM_016061 | Homo sapiens yippee-like 5 (Drosophila) (YPEL5), transcript variant 4, mRNA [NM_016061] | 19.76 |
| NM_138456 | Homo sapiens basic leucine zipper transcription factor, ATF-like 2 (BATF2), mRNA [NM_138456] | 19.74 |
| NM_000930 | Homo sapiens plasminogen activator, tissue (PLAT), transcript variant 1, mRNA [NM_000930] | 19.51 |
| NM_016303 | Homo sapiens WW domain binding protein 5 (WBP5), transcript variant 1, mRNA [NM_016303] | 19.51 |
| NM_018622 | Homo sapiens presenilin associated, rhomboid-like (PARL), nuclear gene encoding mitochondrial protein, transcript variant 1, mRNA [NM_018622] | 19.50 |
| NM_006415 | Homo sapiens serine palmitoyltransferase, long chain base subunit 1 (SPTLC1), transcript variant 1, mRNA [NM_006415] | 19.46 |

| | | |
|---|---|---|
| NM_001024209 | Homo sapiens small proline-rich protein 2E (SPRR2E), mRNA [NM_001024209] | 19.42 |
| NM_006803 | Homo sapiens adaptor-related protein complex 3, mu 2 subunit (AP3M2), transcript variant 2, mRNA [NM_006803] | 19.33 |
| NM_001145373 | Homo sapiens OTU domain containing 1 (OTUD1), mRNA [NM_001145373] | 19.25 |
| NM_032985 | Homo sapiens Sec23 homolog B (S. cerevisiae) (SEC23B), transcript variant 2, mRNA [NM_032985] | 19.16 |
| NM_015172 | Homo sapiens proline-rich coiled-coil 2C (PRRC2C), mRNA [NM_015172] | 19.03 |
| NM_015387 | Homo sapiens MOB1, Mps One Binder kinase activator-like 3 (yeast) (MOBKL3), transcript variant 1, mRNA [NM_015387] | 19.02 |
| NM_033292 | Homo sapiens caspase 1, apoptosis-related cysteine peptidase (interleukin 1, beta, convertase) (CASP1), transcript variant alpha, mRNA [NM_033292] | 19.00 |
| NM_005977 | Homo sapiens ring finger protein (C3H2C3 type) 6 (RNF6), transcript variant 1, mRNA [NM_005977] | 19.00 |
| NM_025137 | Homo sapiens spastic paraplegia 11 (autosomal recessive) (SPG11), transcript variant 1, mRNA [NM_025137] | 18.96 |
| NM_001549 | Homo sapiens interferon-induced protein with tetratricopeptide repeats 3 (IFIT3), transcript variant 1, mRNA [NM_001549] | 18.88 |
| NM_005627 | Homo sapiens serum/glucocorticoid regulated kinase 1 (SGK1), transcript variant 1, mRNA [NM_005627] | 18.83 |
| NM_001011713 | Homo sapiens N(alpha)-acetyltransferase 30, NatC catalytic subunit (NAA30), mRNA [NM_001011713] | 18.80 |
| NM_002709 | Homo sapiens protein phosphatase 1, catalytic subunit, beta isozyme (PPP1CB), transcript variant 1, mRNA [NM_002709] | 18.73 |
| NM_007372 | Homo sapiens DEAD (Asp-Glu-Ala-Asp) box polypeptide 42 (DDX42), transcript variant 1, mRNA [NM_007372] | 18.73 |
| NM_002974 | Homo sapiens serpin peptidase inhibitor, clade B (ovalbumin), member 4 (SERPINB4), mRNA [NM_002974] | 18.73 |
| NM_000361 | Homo sapiens thrombomodulin (THBD), mRNA [NM_000361] | 18.40 |
| NM_001013442 | Homo sapiens epithelial mitogen homolog (mouse) (EPGN), mRNA [NM_001013442] | 18.39 |
| NM_016113 | Homo sapiens transient receptor potential cation channel, subfamily V, member 2 (TRPV2), mRNA [NM_016113] | 18.20 |
| NM_003115 | Homo sapiens UDP-N-acteylglucosamine pyrophosphorylase 1 (UAP1), mRNA [NM_003115] | 18.13 |
| NM_003810 | Homo sapiens tumor necrosis factor (ligand) superfamily, member 10 (TNFSF10), transcript variant 1, mRNA [NM_003810] | 18.10 |
| NM_032521 | Homo sapiens par-6 partitioning defective 6 homolog beta (C. elegans) (PARD6B), mRNA [NM_032521] | 18.09 |
| NM_021214 | Homo sapiens family with sequence similarity 108, member C1 (FAM108C1), mRNA [NM_021214] | 18.04 |
| NM_002526 | Homo sapiens 5′-nucleotidase, ecto (CD73) (NT5E), mRNA [NM_002526] | 18.02 |

| | | |
|---|---|---|
| NM_033641 | Homo sapiens collagen, type IV, alpha 6 (COL4A6), transcript variant B, mRNA [NM_033641] | 18.02 |
| NM_017801 | Homo sapiens CKLF-like MARVEL transmembrane domain containing 6 (CMTM6), mRNA [NM_017801] | 17.86 |
| NM_020821 | Homo sapiens vacuolar protein sorting 13 homolog C (S. cerevisiae) (VPS13C), transcript variant 2A, mRNA [NM_020821] | 17.85 |
| NM_148170 | Homo sapiens cathepsin C (CTSC), transcript variant 2, mRNA [NM_148170] | 17.81 |
| NM_001008493 | Homo sapiens enabled homolog (Drosophila) (ENAH), transcript variant 1, mRNA [NM_001008493] | 17.49 |
| NM_002422 | Homo sapiens matrix metallopeptidase 3 (stromelysin 1, progelatinase) (MMP3), mRNA [NM_002422] | 17.41 |
| NM_173086 | Homo sapiens keratin 6C (KRT6C), mRNA [NM_173086] | 17.33 |
| NM_183005 | Homo sapiens ribonuclease P/MRP 38kDa subunit (RPP38), transcript variant 1, mRNA [NM_183005] | 17.32 |
| NM_001663 | Homo sapiens ADP-ribosylation factor 6 (ARF6), mRNA [NM_001663] | 17.25 |
| NM_002710 | Homo sapiens protein phosphatase 1, catalytic subunit, gamma isozyme (PPP1CC), mRNA [NM_002710] | 17.13 |
| NM_152703 | Homo sapiens sterile alpha motif domain containing 9-like (SAMD9L), mRNA [NM_152703] | 17.04 |
| NM_002835 | Homo sapiens protein tyrosine phosphatase, non-receptor type 12 (PTPN12), transcript variant 1, mRNA [NM_002835] | 17.04 |
| NM_000214 | Homo sapiens jagged 1 (JAG1), mRNA [NM_000214] | 16.94 |
| NM_001001669 | Homo sapiens Rho guanine nucleotide exchange factor (GEF) 37 (ARHGEF37), mRNA [NM_001001669] | 16.91 |
| NM_018947 | Homo sapiens cytochrome c, somatic (CYCS), nuclear gene encoding mitochondrial protein, mRNA [NM_018947] | 16.91 |
| NM_000610 | Homo sapiens CD44 molecule (Indian blood group) (CD44), transcript variant 1, mRNA [NM_000610] | 16.85 |
| NM_016078 | Homo sapiens family with sequence similarity 18, member B1 (FAM18B1), mRNA [NM_016078] | 16.85 |
| NM_033394 | Homo sapiens tetratricopeptide repeat, ankyrin repeat and coiled-coil containing 1 (TANC1), transcript variant 1, mRNA [NM_033394] | 16.81 |
| NM_018948 | Homo sapiens ERBB receptor feedback inhibitor 1 (ERRFI1), mRNA [NM_018948] | 16.72 |
| NM_058172 | Homo sapiens anthrax toxin receptor 2 (ANTXR2), transcript variant 1, mRNA [NM_058172] | 16.71 |
| NM_016626 | Homo sapiens mex-3 homolog C (C. elegans) (MEX3C), mRNA [NM_016626] | 16.68 |
| NM_006372 | Homo sapiens synaptotagmin binding, cytoplasmic RNA interacting protein (SYNCRIP), transcript variant 1, mRNA [NM_006372] | 16.52 |
| NM_004730 | Homo sapiens eukaryotic translation termination factor 1 (ETF1), mRNA [NM_004730] | 16.39 |
| NM_000775 | Homo sapiens cytochrome P450, family 2, subfamily J, polypeptide 2 (CYP2J2), mRNA [NM_000775] | 16.33 |

| | | |
|---|---|---|
| NM_015239 | Homo sapiens ATP/GTP binding protein 1 (AGTPBP1), mRNA [NM_015239] | 16.29 |
| NM_001755 | Homo sapiens core-binding factor, beta subunit (CBFB), transcript variant 2, mRNA [NM_001755] | 16.26 |
| NM_032027 | Homo sapiens TM2 domain containing 1 (TM2D1), mRNA [NM_032027] | 16.22 |
| NM_004550 | Homo sapiens NADH dehydrogenase (ubiquinone) Fe-S protein 2, 49kDa (NADH-coenzyme Q reductase) (NDUFS2), nuclear gene encoding mitochondrial protein, transcript variant 1, mRNA [NM_004550] | 16.22 |
| NM_153045 | Homo sapiens chromosome 9 open reading frame 91 (C9orf91), mRNA [NM_153045] | 16.20 |
| NM_006241 | Homo sapiens protein phosphatase 1, regulatory (inhibitor) subunit 2 (PPP1R2), mRNA [NM_006241] | 16.19 |
| NM_181701 | Homo sapiens quiescin Q6 sulfhydryl oxidase 2 (QSOX2), mRNA [NM_181701] | 16.16 |
| NM_005564 | Homo sapiens lipocalin 2 (LCN2), mRNA [NM_005564] | 16.15 |
| NM_005557 | Homo sapiens keratin 16 (KRT16), mRNA [NM_005557] | 16.12 |
| NM_006310 | Homo sapiens aminopeptidase puromycin sensitive (NPEPPS), mRNA [NM_006310] | 16.10 |
| NM_012242 | Homo sapiens dickkopf homolog 1 (Xenopus laevis) (DKK1), mRNA [NM_012242] | 16.08 |
| NM_005416 | Homo sapiens small proline-rich protein 3 (SPRR3), transcript variant 1, mRNA [NM_005416] | 16.04 |
| NM_000785 | Homo sapiens cytochrome P450, family 27, subfamily B, polypeptide 1 (CYP27B1), nuclear gene encoding mitochondrial protein, mRNA [NM_000785] | 16.03 |
| NM_017813 | Homo sapiens inositol monophosphatase domain containing 1 (IMPAD1), mRNA [NM_017813] | 15.97 |
| NM_006698 | Homo sapiens bladder cancer associated protein (BLCAP), transcript variant 1, mRNA [NM_006698] | 15.92 |
| NM_005130 | Homo sapiens fibroblast growth factor binding protein 1 (FGFBP1), mRNA [NM_005130] | 15.82 |
| NM_007190 | Homo sapiens SEC23 interacting protein (SEC23IP), mRNA [NM_007190] | 15.81 |
| NM_006636 | Homo sapiens methylenetetrahydrofolate dehydrogenase (NADP+ dependent) 2, methenyltetrahydrofolate cyclohydrolase (MTHFD2), nuclear gene encoding mitochondrial protein, transcript variant 1, mRNA [NM_006636] | 15.79 |
| NM_030581 | Homo sapiens WD repeat domain 59 (WDR59), mRNA [NM_030581] | 15.77 |
| NM_005842 | Homo sapiens sprouty homolog 2 (Drosophila) (SPRY2), mRNA [NM_005842] | 15.71 |
| NM_015920 | Homo sapiens ribosomal protein S27-like (RPS27L), mRNA [NM_015920] | 15.70 |
| NM_173843 | Homo sapiens interleukin 1 receptor antagonist (IL1RN), transcript variant 4, mRNA [NM_173843] | 15.58 |

| | | |
|---|---|---|
| NM_144664 | Homo sapiens family with sequence similarity 76, member B (FAM76B), mRNA [NM_144664] | 15.55 |
| NM_022147 | Homo sapiens receptor (chemosensory) transporter protein 4 (RTP4), mRNA [NM_022147] | 15.53 |
| NM_003594 | Homo sapiens transcription termination factor, RNA polymerase II (TTF2), mRNA [NM_003594] | 15.51 |
| NM_020645 | Homo sapiens nuclear receptor interacting protein 3 (NRIP3), mRNA [NM_020645] | 15.50 |
| NM_057749 | Homo sapiens cyclin E2 (CCNE2), mRNA [NM_057749] | 15.50 |
| NM_002463 | Homo sapiens myxovirus (influenza virus) resistance 2 (mouse) (MX2), mRNA [NM_002463] | 15.44 |
| NM_001114 | Homo sapiens adenylate cyclase 7 (ADCY7), mRNA [NM_001114] | 15.43 |
| NM_004419 | Homo sapiens dual specificity phosphatase 5 (DUSP5), mRNA [NM_004419] | 15.43 |
| NM_032320 | Homo sapiens BTB (POZ) domain containing 10 (BTBD10), mRNA [NM_032320] | 15.43 |
| NM_003805 | Homo sapiens CASP2 and RIPK1 domain containing adaptor with death domain (CRADD), mRNA [NM_003805] | 15.37 |
| NM_006520 | Homo sapiens dynein, light chain, Tctex-type 3 (DYNLT3), mRNA [NM_006520] | 15.27 |
| NM_016218 | Homo sapiens polymerase (DNA directed) kappa (POLK), mRNA [NM_016218] | 15.24 |
| NM_000380 | Homo sapiens xeroderma pigmentosum, complementation group A (XPA), transcript variant 1, mRNA [NM_000380] | 15.24 |
| NM_014633 | Homo sapiens Ctr9, Paf1/RNA polymerase II complex component, homolog (S. cerevisiae) (CTR9), mRNA [NM_014633] | 15.24 |
| NM_001698 | Homo sapiens AU RNA binding protein/enoyl-CoA hydratase (AUH), nuclear gene encoding mitochondrial protein, mRNA [NM_001698] | 15.24 |
| NM_152444 | Homo sapiens prostaglandin reductase 2 (PTGR2), transcript variant 1, mRNA [NM_152444] | 15.23 |
| NM_144591 | Homo sapiens chromosome 10 open reading frame 32 (C10orf32), transcript variant 2, mRNA [NM_144591] | 15.22 |
| NM_016227 | Homo sapiens chromosome 1 open reading frame 9 (C1orf9), transcript variant 2, mRNA [NM_016227] | 15.11 |
| NM_002392 | Homo sapiens Mdm2 p53 binding protein homolog (mouse) (MDM2), transcript variant MDM2, mRNA [NM_002392] | 15.08 |
| NM_012137 | Homo sapiens dimethylarginine dimethylaminohydrolase 1 (DDAH1), transcript variant 1, mRNA [NM_012137] | 15.08 |
| NM_138773 | Homo sapiens solute carrier family 25, member 46 (SLC25A46), mRNA [NM_138773] | 15.06 |
| NM_020199 | Homo sapiens chromosome 5 open reading frame 15 (C5orf15), mRNA [NM_020199] | 15.06 |
| NM_016824 | Homo sapiens adducin 3 (gamma) (ADD3), transcript variant 1, mRNA [NM_016824] | 15.05 |
| NM_020357 | Homo sapiens PEST proteolytic signal containing nuclear protein (PCNP), mRNA [NM_020357] | 14.98 |

| | | |
|---|---|---|
| NM_016464 | Homo sapiens transmembrane protein 138 (TMEM138), transcript variant 1, mRNA [NM_016464] | 14.91 |
| NM_006933 | Homo sapiens solute carrier family 5 (sodium/myo-inositol cotransporter), member 3 (SLC5A3), mRNA [NM_006933] | 14.87 |
| NM_018374 | Homo sapiens transmembrane protein 106B (TMEM106B), transcript variant 1, mRNA [NM_018374] | 14.87 |
| NM_001023571 | Homo sapiens IQ motif containing B1 (IQCB1), transcript variant 3, mRNA [NM_001023571] | 14.85 |
| NM_016308 | Homo sapiens cytidine monophosphate (UMP-CMP) kinase 1, cytosolic (CMPK1), transcript variant 1, mRNA [NM_016308] | 14.79 |
| NM_002572 | Homo sapiens platelet-activating factor acetylhydrolase 1b, catalytic subunit 2 (30kDa) (PAFAH1B2), transcript variant 1, mRNA [NM_002572] | 14.78 |
| NM_002078 | Homo sapiens golgin A4 (GOLGA4), transcript variant 2, mRNA [NM_002078] | 14.72 |
| NM_020240 | Homo sapiens CDC42 small effector 2 (CDC42SE2), transcript variant 1, mRNA [NM_020240] | 14.67 |
| NM_015423 | Homo sapiens aminoadipate-semialdehyde dehydrogenase-phosphopantetheinyl transferase (AASDHPPT), mRNA [NM_015423] | 14.64 |
| NM_024725 | Homo sapiens coiled-coil domain containing 82 (CCDC82), mRNA [NM_024725] | 14.58 |
| NM_015176 | Homo sapiens F-box protein 28 (FBXO28), transcript variant 1, mRNA [NM_015176] | 14.57 |
| NM_006417 | Homo sapiens interferon-induced protein 44 (IFI44), mRNA [NM_006417] | 14.55 |
| NM_001039479 | Homo sapiens KIAA0317 (KIAA0317), mRNA [NM_001039479] | 14.51 |
| NM_006793 | Homo sapiens peroxiredoxin 3 (PRDX3), nuclear gene encoding mitochondrial protein, transcript variant 1, mRNA [NM_006793] | 14.50 |
| NM_033426 | Homo sapiens KIAA1737 (KIAA1737), mRNA [NM_033426] | 14.48 |
| NM_032034 | Homo sapiens solute carrier family 4, sodium borate transporter, member 11 (SLC4A11), transcript variant 2, mRNA [NM_032034] | 14.48 |
| NM_014398 | Homo sapiens lysosomal-associated membrane protein 3 (LAMP3), mRNA [NM_014398] | 14.44 |
| NM_024039 | Homo sapiens MIS12, MIND kinetochore complex component, homolog (S. pombe) (MIS12), mRNA [NM_024039] | 14.37 |
| NM_014720 | Homo sapiens STE20-like kinase (SLK), mRNA [NM_014720] | 14.37 |
| NM_000693 | Homo sapiens aldehyde dehydrogenase 1 family, member A3 (ALDH1A3), mRNA [NM_000693] | 14.33 |
| NM_015534 | Homo sapiens zinc finger, ZZ-type containing 3 (ZZZ3), mRNA [NM_015534] | 14.27 |
| NM_000147 | Homo sapiens fucosidase, alpha-L- 1, tissue (FUCA1), mRNA [NM_000147] | 14.26 |
| NM_004124 | Homo sapiens glia maturation factor, beta (GMFB), mRNA [NM_004124] | 14.26 |
| NM_003020 | Homo sapiens secretogranin V (7B2 protein) (SCG5), transcript variant 2, mRNA [NM_003020] | 14.25 |

| | | |
|---|---|---|
| NM_032557 | Homo sapiens ubiquitin specific peptidase 38 (USP38), mRNA [NM_032557] | 14.22 |
| NM_012129 | Homo sapiens claudin 12 (CLDN12), transcript variant 3, mRNA [NM_012129] | 14.12 |
| NM_153485 | Homo sapiens nucleoporin 155kDa (NUP155), transcript variant 1, mRNA [NM_153485] | 14.12 |
| NM_001621 | Homo sapiens aryl hydrocarbon receptor (AHR), mRNA [NM_001621] | 14.11 |
| NM_014962 | Homo sapiens BTB (POZ) domain containing 3 (BTBD3), transcript variant 1, mRNA [NM_014962] | 14.10 |
| NM_016323 | Homo sapiens hect domain and RLD 5 (HERC5), mRNA [NM_016323] | 14.09 |
| NM_024835 | Homo sapiens gametogenetin binding protein 2 (GGNBP2), mRNA [NM_024835] | 14.09 |
| NM_031453 | Homo sapiens family with sequence similarity 107, member B (FAM107B), mRNA [NM_031453] | 14.06 |
| NM_001017395 | Homo sapiens transmembrane and coiled-coil domain family 1 (TMCC1), transcript variant 1, mRNA [NM_001017395] | 14.03 |
| NM_005168 | Homo sapiens Rho family GTPase 3 (RND3), mRNA [NM_005168] | 14.02 |
| NM_001007245 | Homo sapiens interferon-related developmental regulator 1 (IFRD1), transcript variant 2, mRNA [NM_001007245] | 13.97 |
| NM_032390 | Homo sapiens MKI67 (FHA domain) interacting nucleolar phosphoprotein (MKI67IP), mRNA [NM_032390] | 13.86 |
| NM_020899 | Homo sapiens zinc finger and BTB domain containing 4 (ZBTB4), transcript variant 1, mRNA [NM_020899] | 13.81 |
| NM_198194 | Homo sapiens stomatin (STOM), transcript variant 2, mRNA [NM_198194] | 13.78 |
| NM_033109 | Homo sapiens polyribonucleotide nucleotidyltransferase 1 (PNPT1), mRNA [NM_033109] | 13.77 |
| NM_004136 | Homo sapiens iron-responsive element binding protein 2 (IREB2), mRNA [NM_004136] | 13.75 |
| NM_021218 | Homo sapiens chromosome 9 open reading frame 80 (C9orf80), mRNA [NM_021218] | 13.74 |
| NM_005426 | Homo sapiens tumor protein p53 binding protein, 2 (TP53BP2), transcript variant 2, mRNA [NM_005426] | 13.74 |
| NM_021252 | Homo sapiens RAB18, member RAS oncogene family (RAB18), mRNA [NM_021252] | 13.70 |
| NM_001024847 | Homo sapiens transforming growth factor, beta receptor II (70/80kDa) (TGFBR2), transcript variant 1, mRNA [NM_001024847] | 13.66 |
| NM_016070 | Homo sapiens mitochondrial ribosomal protein S23 (MRPS23), nuclear gene encoding mitochondrial protein, mRNA [NM_016070] | 13.62 |
| NM_153262 | Homo sapiens synaptotagmin XIV (SYT14), transcript variant 4, mRNA [NM_153262] | 13.61 |
| NM_005980 | Homo sapiens S100 calcium binding protein P (S100P), mRNA [NM_005980] | 13.59 |
| NM_172178 | Homo sapiens mitochondrial ribosomal protein L42 (MRPL42), nuclear gene encoding mitochondrial protein, transcript variant 3, mRNA [NM_172178] | 13.59 |

| | | |
|---|---|---|
| NM_006147 | Homo sapiens interferon regulatory factor 6 (IRF6), mRNA [NM_006147] | 13.57 |
| NM_020424 | Homo sapiens LYR motif containing 1 (LYRM1), transcript variant 1, mRNA [NM_020424] | 13.56 |
| NM_182491 | Homo sapiens zinc finger, AN1-type domain 2A (ZFAND2A), mRNA [NM_182491] | 13.52 |
| NM_019600 | Homo sapiens KIAA1370 (KIAA1370), mRNA [NM_019600] | 13.52 |
| NM_033285 | Homo sapiens tumor protein p53 inducible nuclear protein 1 (TP53INP1), transcript variant 1, mRNA [NM_033285] | 13.50 |
| NM_001033503 | Homo sapiens SAR1 homolog B (S. cerevisiae) (SAR1B), transcript variant 1, mRNA [NM_001033503] | 13.47 |
| NM_001172428 | Homo sapiens kelch-like 7 (Drosophila) (KLHL7), transcript variant 3, mRNA [NM_001172428] | 13.46 |
| NM_014819 | Homo sapiens praja ring finger 2 (PJA2), mRNA [NM_014819] | 13.41 |
| NM_014321 | Homo sapiens origin recognition complex, subunit 6 (ORC6), transcript variant 1, mRNA [NM_014321] | 13.39 |
| NM_194328 | Homo sapiens ring finger protein 38 (RNF38), transcript variant 2, mRNA [NM_194328] | 13.37 |
| NM_002639 | Homo sapiens serpin peptidase inhibitor, clade B (ovalbumin), member 5 (SERPINB5), mRNA [NM_002639] | 13.37 |
| NM_001032293 | Homo sapiens zinc finger protein 207 (ZNF207), transcript variant 2, mRNA [NM_001032293] | 13.37 |
| NM_014866 | Homo sapiens SEC16 homolog A (S. cerevisiae) (SEC16A), mRNA [NM_014866] | 13.34 |
| NM_032558 | Homo sapiens hippocampus abundant transcript-like 1 (HIATL1), mRNA [NM_032558] | 13.34 |
| NM_015713 | Homo sapiens ribonucleotide reductase M2 B (TP53 inducible) (RRM2B), transcript variant 1, mRNA [NM_015713] | 13.32 |
| NM_080652 | Homo sapiens transmembrane protein 41A (TMEM41A), mRNA [NM_080652] | 13.30 |
| NM_016531 | Homo sapiens Kruppel-like factor 3 (basic) (KLF3), mRNA [NM_016531] | 13.29 |
| NM_002638 | Homo sapiens peptidase inhibitor 3, skin-derived (PI3), mRNA [NM_002638] | 13.29 |
| NM_025185 | Homo sapiens tetratricopeptide repeat, ankyrin repeat and coiled-coil containing 2 (TANC2), mRNA [NM_025185] | 13.28 |
| NM_001034116 | Homo sapiens eukaryotic translation initiation factor 2B, subunit 4 delta, 67kDa (EIF2B4), transcript variant 2, mRNA [NM_001034116] | 13.26 |
| NM_013448 | Homo sapiens bromodomain adjacent to zinc finger domain, 1A (BAZ1A), transcript variant 1, mRNA [NM_013448] | 13.25 |
| NM_170695 | Homo sapiens TGFB-induced factor homeobox 1 (TGIF1), transcript variant 1, mRNA [NM_170695] | 13.23 |
| NM_138782 | Homo sapiens FCH domain only 2 (FCHO2), transcript variant 1, mRNA [NM_138782] | 13.23 |
| NM_178454 | Homo sapiens DNA-damage regulated autophagy modulator 2 (DRAM2), mRNA [NM_178454] | 13.18 |

| | | |
|---|---|---|
| NM_030799 | Homo sapiens Yip1 domain family, member 5 (YIPF5), transcript variant 2, mRNA [NM_030799] | 13.17 |
| NM_018976 | Homo sapiens solute carrier family 38, member 2 (SLC38A2), mRNA [NM_018976] | 13.17 |
| NM_003264 | Homo sapiens toll-like receptor 2 (TLR2), mRNA [NM_003264] | 13.15 |
| NM_024573 | Homo sapiens chromosome 6 open reading frame 211 (C6orf211), mRNA [NM_024573] | 13.13 |
| NM_003794 | Homo sapiens sorting nexin 4 (SNX4), mRNA [NM_003794] | 13.08 |
| NM_004890 | Homo sapiens sperm associated antigen 7 (SPAG7), mRNA [NM_004890] | 13.07 |
| NM_152829 | Homo sapiens testis derived transcript (3 LIM domains) (TES), transcript variant 2, mRNA [NM_152829] | 13.04 |
| NM_002125 | Homo sapiens major histocompatibility complex, class II, DR beta 5 (HLA-DRB5), mRNA [NM_002125] | 13.02 |
| NM_005536 | Homo sapiens inositol(myo)-1(or 4)-monophosphatase 1 (IMPA1), transcript variant 1, mRNA [NM_005536] | 12.99 |
| NM_014423 | Homo sapiens AF4/FMR2 family, member 4 (AFF4), mRNA [NM_014423] | 12.99 |
| NM_006537 | Homo sapiens ubiquitin specific peptidase 3 (USP3), mRNA [NM_006537] | 12.93 |
| NM_014793 | Homo sapiens leucine carboxyl methyltransferase 2 (LCMT2), mRNA [NM_014793] | 12.93 |
| NM_022483 | Homo sapiens chromosome 5 open reading frame 28 (C5orf28), mRNA [NM_022483] | 12.90 |
| NM_021105 | Homo sapiens phospholipid scramblase 1 (PLSCR1), mRNA [NM_021105] | 12.86 |
| NM_018330 | Homo sapiens KIAA1598 (KIAA1598), transcript variant 2, mRNA [NM_018330] | 12.86 |
| NM_015143 | Homo sapiens methionyl aminopeptidase 1 (METAP1), mRNA [NM_015143] | 12.85 |
| NM_012434 | Homo sapiens solute carrier family 17 (anion/sugar transporter), member 5 (SLC17A5), mRNA [NM_012434] | 12.84 |
| NM_018269 | Homo sapiens acireductone dioxygenase 1 (ADI1), mRNA [NM_018269] | 12.83 |
| NM_012142 | Homo sapiens cyclin D-type binding-protein 1 (CCNDBP1), transcript variant 1, mRNA [NM_012142] | 12.83 |
| NM_015257 | Homo sapiens transmembrane protein 194A (TMEM194A), transcript variant 2, mRNA [NM_015257] | 12.80 |
| NM_021140 | Homo sapiens lysine (K)-specific demethylase 6A (KDM6A), mRNA [NM_021140] | 12.78 |
| NM_000700 | Homo sapiens annexin A1 (ANXA1), mRNA [NM_000700] | 12.67 |
| NM_018112 | Homo sapiens transmembrane protein 38B (TMEM38B), mRNA [NM_018112] | 12.66 |
| NM_017610 | Homo sapiens ring finger protein 111 (RNF111), mRNA [NM_017610] | 12.65 |
| NM_002210 | Homo sapiens integrin, alpha V (vitronectin receptor, alpha polypeptide, antigen CD51) (ITGAV), transcript variant 1, mRNA [NM_002210] | 12.63 |

| | | |
|---|---|---|
| NM_006996 | Homo sapiens solute carrier family 19 (thiamine transporter), member 2 (SLC19A2), mRNA [NM_006996] | 12.63 |
| NM_005667 | Homo sapiens ring finger protein 103 (RNF103), transcript variant 1, mRNA [NM_005667] | 12.63 |
| NM_001957 | Homo sapiens endothelin receptor type A (EDNRA), transcript variant 1, mRNA [NM_001957] | 12.61 |
| NM_000271 | Homo sapiens Niemann-Pick disease, type C1 (NPC1), mRNA [NM_000271] | 12.59 |
| NM_006153 | Homo sapiens NCK adaptor protein 1 (NCK1), transcript variant 1, mRNA [NM_006153] | 12.58 |
| NM_018237 | Homo sapiens cell division cycle and apoptosis regulator 1 (CCAR1), mRNA [NM_018237] | 12.57 |
| NM_138768 | Homo sapiens myeloma overexpressed (in a subset of t(11;14) positive multiple myelomas) (MYEOV), mRNA [NM_138768] | 12.55 |
| NM_005113 | Homo sapiens golgin A5 (GOLGA5), mRNA [NM_005113] | 12.54 |
| NM_022121 | Homo sapiens PERP, TP53 apoptosis effector (PERP), mRNA [NM_022121] | 12.54 |
| NM_198182 | Homo sapiens grainyhead-like 1 (Drosophila) (GRHL1), mRNA [NM_198182] | 12.54 |
| NM_012080 | Homo sapiens haloacid dehalogenase-like hydrolase domain containing 1 (HDHD1), transcript variant 2, mRNA [NM_012080] | 12.52 |
| NM_000584 | Homo sapiens interleukin 8 (IL8), mRNA [NM_000584] | 12.50 |
| NM_024683 | Homo sapiens chromosome 17 open reading frame 42 (C17orf42), mRNA [NM_024683] | 12.50 |
| NM_152546 | Homo sapiens serum response factor binding protein 1 (SRFBP1), mRNA [NM_152546] | 12.48 |
| NM_015383 | Homo sapiens neuroblastoma breakpoint family, member 14 (NBPF14), mRNA [NM_015383] | 12.46 |
| NM_001048183 | Homo sapiens phosphatase and actin regulator 4 (PHACTR4), transcript variant 1, mRNA [NM_001048183] | 12.43 |
| NM_001548 | Homo sapiens interferon-induced protein with tetratricopeptide repeats 1 (IFIT1), transcript variant 2, mRNA [NM_001548] | 12.42 |
| NM_024551 | Homo sapiens adiponectin receptor 2 (ADIPOR2), mRNA [NM_024551] | 12.42 |
| NM_145686 | Homo sapiens mitogen-activated protein kinase kinase kinase kinase 4 (MAP4K4), transcript variant 2, mRNA [NM_145686] | 12.41 |
| NM_005402 | Homo sapiens v-ral simian leukemia viral oncogene homolog A (ras related) (RALA), mRNA [NM_005402] | 12.38 |
| NM_021983 | Homo sapiens major histocompatibility complex, class II, DR beta 4 (HLA-DRB4), mRNA [NM_021983] | 12.37 |
| NM_003898 | Homo sapiens synaptojanin 2 (SYNJ2), transcript variant 1, mRNA [NM_003898] | 12.36 |
| NM_014990 | Homo sapiens Ral GTPase activating protein, alpha subunit 1 (catalytic) (RALGAPA1), transcript variant 1, mRNA [NM_014990] | 12.36 |
| NM_000575 | Homo sapiens interleukin 1, alpha (IL1A), mRNA [NM_000575] | 12.35 |

| | | |
|---|---|---|
| NM_001033557 | Homo sapiens protein phosphatase, Mg2+/Mn2+ dependent, 1B (PPM1B), transcript variant 5, mRNA [NM_001033557] | 12.34 |
| NM_002956 | Homo sapiens CAP-GLY domain containing linker protein 1 (CLIP1), transcript variant 1, mRNA [NM_002956] | 12.31 |
| NM_000617 | Homo sapiens solute carrier family 11 (proton-coupled divalent metal ion transporters), member 2 (SLC11A2), transcript variant 4, mRNA [NM_000617] | 12.24 |
| NM_016072 | Homo sapiens golgi transport 1B (GOLT1B), mRNA [NM_016072] | 12.22 |
| NM_153212 | Homo sapiens gap junction protein, beta 4, 30.3kDa (GJB4), mRNA [NM_153212] | 12.21 |
| NM_020820 | Homo sapiens phosphatidylinositol-3,4,5-trisphosphate-dependent Rac exchange factor 1 (PREX1), mRNA [NM_020820] | 12.19 |
| NM_000489 | Homo sapiens alpha thalassemia/mental retardation syndrome X-linked (ATRX), transcript variant 1, mRNA [NM_000489] | 12.10 |
| NM_003311 | Homo sapiens pleckstrin homology-like domain, family A, member 2 (PHLDA2), mRNA [NM_003311] | 12.09 |
| NM_001034 | Homo sapiens ribonucleotide reductase M2 (RRM2), transcript variant 2, mRNA [NM_001034] | 12.06 |
| NM_016097 | Homo sapiens immediate early response 3 interacting protein 1 (IER3IP1), mRNA [NM_016097] | 12.02 |
| NM_015957 | Homo sapiens APAF1 interacting protein (APIP), mRNA [NM_015957] | 11.97 |
| NM_001098783 | Homo sapiens ribonuclease P/MRP 14kDa subunit (RPP14), transcript variant 1, mRNA [NM_001098783] | 11.97 |
| NM_003016 | Homo sapiens serine/arginine-rich splicing factor 2 (SRSF2), transcript variant 1, mRNA [NM_003016] | 11.91 |
| NM_004866 | Homo sapiens secretory carrier membrane protein 1 (SCAMP1), mRNA [NM_004866] | 11.89 |
| NM_001025390 | Homo sapiens adenosine monophosphate deaminase 3 (AMPD3), transcript variant 3, mRNA [NM_001025390] | 11.89 |
| NM_024625 | Homo sapiens zinc finger CCCH-type, antiviral 1 (ZC3HAV1), transcript variant 2, mRNA [NM_024625] | 11.87 |
| NM_001018090 | Homo sapiens GRINL1A complex locus (GCOM1), transcript variant 1, mRNA [NM_001018090] | 11.85 |
| NM_002270 | Homo sapiens transportin 1 (TNPO1), transcript variant 1, mRNA [NM_002270] | 11.84 |
| NM_001178106 | Homo sapiens zinc finger protein 185 (LIM domain) (ZNF185), transcript variant 1, mRNA [NM_001178106] | 11.83 |
| NM_020150 | Homo sapiens SAR1 homolog A (S. cerevisiae) (SAR1A), transcript variant 2, mRNA [NM_020150] | 11.82 |
| NM_080489 | Homo sapiens syndecan binding protein (syntenin) 2 (SDCBP2), transcript variant 1, mRNA [NM_080489] | 11.80 |
| NM_053045 | Homo sapiens transmembrane protein 203 (TMEM203), mRNA [NM_053045] | 11.78 |
| NM_031905 | Homo sapiens armadillo repeat containing 10 (ARMC10), transcript variant A, mRNA [NM_031905] | 11.77 |

| | | |
|---|---|---|
| NM_058238 | Homo sapiens wingless-type MMTV integration site family, member 7B (WNT7B), mRNA [NM_058238] | 11.76 |
| NM_006380 | Homo sapiens amyloid beta precursor protein (cytoplasmic tail) binding protein 2 (APPBP2), mRNA [NM_006380] | 11.76 |
| NM_020830 | Homo sapiens WD repeat and FYVE domain containing 1 (WDFY1), mRNA [NM_020830] | 11.75 |
| NM_006945 | Homo sapiens small proline-rich protein 2D (SPRR2D), mRNA [NM_006945] | 11.73 |
| NM_001143764 | Homo sapiens synaptonemal complex central element protein 1 (SYCE1), transcript variant 4, mRNA [NM_001143764] | 11.69 |
| NM_003907 | Homo sapiens eukaryotic translation initiation factor 2B, subunit 5 epsilon, 82kDa (EIF2B5), mRNA [NM_003907] | 11.68 |
| NM_014970 | Homo sapiens kinesin-associated protein 3 (KIFAP3), mRNA [NM_014970] | 11.65 |
| NM_016548 | Homo sapiens golgi membrane protein 1 (GOLM1), transcript variant 1, mRNA [NM_016548] | 11.65 |
| NM_003922 | Homo sapiens hect (homologous to the E6-AP (UBE3A) carboxyl terminus) domain and RCC1 (CHC1)-like domain (RLD) 1 (HERC1), mRNA [NM_003922] | 11.63 |
| NM_001030273 | Homo sapiens aryl hydrocarbon receptor nuclear translocator-like (ARNTL), transcript variant 3, mRNA [NM_001030273] | 11.62 |
| NM_015328 | Homo sapiens adenosylhomocysteinase-like 2 (AHCYL2), transcript variant 1, mRNA [NM_015328] | 11.62 |
| NM_001002259 | Homo sapiens caprin family member 2 (CAPRIN2), transcript variant 1, mRNA [NM_001002259] | 11.61 |
| NM_002643 | Homo sapiens phosphatidylinositol glycan anchor biosynthesis, class F (PIGF), transcript variant 1, mRNA [NM_002643] | 11.61 |
| NM_003453 | Homo sapiens zinc finger, MYM-type 2 (ZMYM2), transcript variant 1, mRNA [NM_003453] | 11.60 |
| NM_152231 | Homo sapiens F-box protein 34 (FBXO34), transcript variant 2, mRNA [NM_152231] | 11.58 |
| NM_004849 | Homo sapiens ATG5 autophagy related 5 homolog (S. cerevisiae) (ATG5), mRNA [NM_004849] | 11.57 |
| NM_152417 | Homo sapiens transmembrane protein 68 (TMEM68), mRNA [NM_152417] | 11.56 |
| NM_002880 | Homo sapiens v-raf-1 murine leukemia viral oncogene homolog 1 (RAF1), mRNA [NM_002880] | 11.56 |
| NM_005732 | Homo sapiens RAD50 homolog (S. cerevisiae) (RAD50), mRNA [NM_005732] | 11.52 |
| NM_005032 | Homo sapiens plastin 3 (PLS3), transcript variant 1, mRNA [NM_005032] | 11.51 |
| NM_000028 | Homo sapiens amylo-alpha-1, 6-glucosidase, 4-alpha-glucanotransferase (AGL), transcript variant 4, mRNA [NM_000028] | 11.50 |
| NM_001011658 | Homo sapiens trafficking protein particle complex 2 (TRAPPC2), transcript variant 1, mRNA [NM_001011658] | 11.47 |

| | | |
|---|---|---|
| NM_032509 | Homo sapiens MAK16 homolog (S. cerevisiae) (MAK16), mRNA [NM_032509] | 11.45 |
| NM_004569 | Homo sapiens phosphatidylinositol glycan anchor biosynthesis, class H (PIGH), mRNA [NM_004569] | 11.43 |
| NM_007217 | Homo sapiens programmed cell death 10 (PDCD10), transcript variant 1, mRNA [NM_007217] | 11.43 |
| NM_004755 | Homo sapiens ribosomal protein S6 kinase, 90kDa, polypeptide 5 (RPS6KA5), transcript variant 1, mRNA [NM_004755] | 11.41 |
| NM_001660 | Homo sapiens ADP-ribosylation factor 4 (ARF4), mRNA [NM_001660] | 11.41 |
| NM_004161 | Homo sapiens RAB1A, member RAS oncogene family (RAB1A), transcript variant 1, mRNA [NM_004161] | 11.41 |
| NM_019111 | Homo sapiens major histocompatibility complex, class II, DR alpha (HLA-DRA), mRNA [NM_019111] | 11.40 |
| NM_021999 | Homo sapiens integral membrane protein 2B (ITM2B), mRNA [NM_021999] | 11.39 |
| NM_025154 | Homo sapiens Sad1 and UNC84 domain containing 1 (SUN1), transcript variant 2, mRNA [NM_025154] | 11.39 |
| NM_203330 | Homo sapiens CD59 molecule, complement regulatory protein (CD59), transcript variant 1, mRNA [NM_203330] | 11.39 |
| NM_003714 | Homo sapiens stanniocalcin 2 (STC2), mRNA [NM_003714] | 11.37 |
| NM_004705 | Homo sapiens protein-kinase, interferon-inducible double stranded RNA dependent inhibitor, repressor of (P58 repressor) (PRKRIR), mRNA [NM_004705] | 11.36 |
| NM_005875 | Homo sapiens eukaryotic translation initiation factor 1B (EIF1B), mRNA [NM_005875] | 11.33 |
| NM_002849 | Homo sapiens protein tyrosine phosphatase, receptor type, R (PTPRR), transcript variant 1, mRNA [NM_002849] | 11.30 |
| NM_015358 | Homo sapiens MORC family CW-type zinc finger 3 (MORC3), mRNA [NM_015358] | 11.28 |
| NM_001025100 | Homo sapiens myelin basic protein (MBP), transcript variant 8, mRNA [NM_001025100] | 11.27 |
| NM_001950 | Homo sapiens E2F transcription factor 4, p107/p130-binding (E2F4), mRNA [NM_001950] | 11.26 |
| NM_006784 | Homo sapiens WD repeat domain 3 (WDR3), mRNA [NM_006784] | 11.19 |
| NM_203505 | Homo sapiens GTPase activating protein (SH3 domain) binding protein 2 (G3BP2), transcript variant 1, mRNA [NM_203505] | 11.17 |
| NM_017867 | Homo sapiens chromosome 4 open reading frame 27 (C4orf27), mRNA [NM_017867] | 11.17 |
| NM_020705 | Homo sapiens TBC1 domain family, member 24 (TBC1D24), transcript variant 2, mRNA [NM_020705] | 11.16 |
| NM_009587 | Homo sapiens lectin, galactoside-binding, soluble, 9 (LGALS9), transcript variant 1, mRNA [NM_009587] | 11.15 |
| NM_006135 | Homo sapiens capping protein (actin filament) muscle Z-line, alpha 1 (CAPZA1), mRNA [NM_006135] | 11.14 |
| NM_006527 | Homo sapiens stem-loop binding protein (SLBP), mRNA [NM_006527] | 11.13 |

| | | |
|---|---|---|
| NM_024595 | Homo sapiens akirin 1 (AKIRIN1), transcript variant 1, mRNA [NM_024595] | 11.11 |
| NM_014708 | Homo sapiens kinetochore associated 1 (KNTC1), mRNA [NM_014708] | 11.10 |
| NM_032802 | Homo sapiens signal peptide peptidase-like 2A (SPPL2A), mRNA [NM_032802] | 11.10 |
| NM_018180 | Homo sapiens DEAH (Asp-Glu-Ala-His) box polypeptide 32 (DHX32), mRNA [NM_018180] | 11.09 |
| NM_001080530 | Homo sapiens sorting nexin 29 (SNX29), mRNA [NM_001080530] | 11.07 |
| NM_153354 | Homo sapiens transmembrane protein 161B (TMEM161B), mRNA [NM_153354] | 11.07 |
| NM_133443 | Homo sapiens glutamic pyruvate transaminase (alanine aminotransferase) 2 (GPT2), transcript variant 1, mRNA [NM_133443] | 11.06 |
| NM_006777 | Homo sapiens zinc finger and BTB domain containing 33 (ZBTB33), transcript variant 2, mRNA [NM_006777] | 11.05 |
| NM_024546 | Homo sapiens ring finger protein 219 (RNF219), mRNA [NM_024546] | 11.04 |
| NM_080678 | Homo sapiens ubiquitin-conjugating enzyme E2F (putative) (UBE2F), mRNA [NM_080678] | 11.03 |
| NM_002298 | Homo sapiens lymphocyte cytosolic protein 1 (L-plastin) (LCP1), mRNA [NM_002298] | 11.03 |
| NM_016297 | Homo sapiens prenylcysteine oxidase 1 (PCYOX1), mRNA [NM_016297] | 11.02 |
| NM_014143 | Homo sapiens CD274 molecule (CD274), mRNA [NM_014143] | 11.02 |
| NM_005859 | Homo sapiens purine-rich element binding protein A (PURA), mRNA [NM_005859] | 11.00 |
| NM_014345 | Homo sapiens zinc finger protein 318 (ZNF318), mRNA [NM_014345] | 10.98 |
| NM_016578 | Homo sapiens remodeling and spacing factor 1 (RSF1), mRNA [NM_016578] | 10.98 |
| NM_001037225 | Homo sapiens chromosome 11 open reading frame 85 (C11orf85), mRNA [NM_001037225] | 10.98 |
| NM_015485 | Homo sapiens RWD domain containing 3 (RWDD3), transcript variant 1, mRNA [NM_015485] | 10.94 |
| NM_001126 | Homo sapiens adenylosuccinate synthase (ADSS), mRNA [NM_001126] | 10.93 |
| NM_001012964 | Homo sapiens kallikrein-related peptidase 6 (KLK6), transcript variant B, mRNA [NM_001012964] | 10.92 |
| NM_019008 | Homo sapiens Smith-Magenis syndrome chromosome region, candidate 7-like (SMCR7L), mRNA [NM_019008] | 10.92 |
| NM_153706 | Homo sapiens chromosome 5 open reading frame 35 (C5orf35), transcript variant 1, mRNA [NM_153706] | 10.91 |
| NM_004864 | Homo sapiens growth differentiation factor 15 (GDF15), mRNA [NM_004864] | 10.90 |
| NM_001033112 | Homo sapiens poly(A) binding protein interacting protein 2 (PAIP2), transcript variant 1, mRNA [NM_001033112] | 10.90 |
| NM_002222 | Homo sapiens inositol 1,4,5-triphosphate receptor, type 1 (ITPR1), transcript variant 2, mRNA [NM_002222] | 10.88 |

| | | |
|---|---|---|
| NM_022776 | Homo sapiens oxysterol binding protein-like 11 (OSBPL11), mRNA [NM_022776] | 10.87 |
| NM_138714 | Homo sapiens nuclear factor of activated T-cells 5, tonicity-responsive (NFAT5), transcript variant 1, mRNA [NM_138714] | 10.85 |
| NM_001100623 | Homo sapiens pleckstrin homology domain containing, family B (evectins) member 2 (PLEKHB2), transcript variant 3, mRNA [NM_001100623] | 10.85 |
| NM_003185 | Homo sapiens TAF4 RNA polymerase II, TATA box binding protein (TBP)-associated factor, 135kDa (TAF4), mRNA [NM_003185] | 10.84 |
| NM_213590 | Homo sapiens tripartite motif-containing 13 (TRIM13), transcript variant 3, mRNA [NM_213590] | 10.84 |
| NM_178865 | Homo sapiens serine incorporator 2 (SERINC2), transcript variant 1, mRNA [NM_178865] | 10.81 |
| NM_005896 | Homo sapiens isocitrate dehydrogenase 1 (NADP+), soluble (IDH1), mRNA [NM_005896] | 10.81 |
| NM_004622 | Homo sapiens translin (TSN), mRNA [NM_004622] | 10.81 |
| NM_153374 | Homo sapiens LysM, putative peptidoglycan-binding, domain containing 2 (LYSMD2), transcript variant 1, mRNA [NM_153374] | 10.80 |
| NM_030752 | Homo sapiens t-complex 1 (TCP1), transcript variant 1, mRNA [NM_030752] | 10.80 |
| NM_145693 | Homo sapiens lipin 1 (LPIN1), mRNA [NM_145693] | 10.79 |
| NM_000436 | Homo sapiens 3-oxoacid CoA transferase 1 (OXCT1), nuclear gene encoding mitochondrial protein, mRNA [NM_000436] | 10.76 |
| NM_014500 | Homo sapiens HIV-1 Tat specific factor 1 (HTATSF1), transcript variant 2, mRNA [NM_014500] | 10.76 |
| NM_022366 | Homo sapiens transcription factor B2, mitochondrial (TFB2M), nuclear gene encoding mitochondrial protein, mRNA [NM_022366] | 10.76 |
| NM_018137 | Homo sapiens protein arginine methyltransferase 6 (PRMT6), mRNA [NM_018137] | 10.75 |
| NM_021215 | Homo sapiens regulation of nuclear pre-mRNA domain containing 1B (RPRD1B), mRNA [NM_021215] | 10.72 |
| NM_001967 | Homo sapiens eukaryotic translation initiation factor 4A2 (EIF4A2), mRNA [NM_001967] | 10.71 |
| NM_182752 | Homo sapiens tumor protein p63 regulated 1-like (TPRG1L), mRNA [NM_182752] | 10.71 |
| NM_020192 | Homo sapiens chromosome 7 open reading frame 36 (C7orf36), mRNA [NM_020192] | 10.70 |
| NM_053024 | Homo sapiens profilin 2 (PFN2), transcript variant 1, mRNA [NM_053024] | 10.68 |
| NM_138462 | Homo sapiens zinc finger, MYND-type containing 19 (ZMYND19), mRNA [NM_138462] | 10.68 |
| NM_018999 | Homo sapiens family with sequence similarity 190, member B (FAM190B), mRNA [NM_018999] | 10.67 |
| NM_006333 | Homo sapiens C1D nuclear receptor corepressor (C1D), transcript variant 1, mRNA [NM_006333] | 10.67 |

| | | |
|---|---|---|
| NM_153607 | Homo sapiens chromosome 5 open reading frame 41 (C5orf41), transcript variant 1, mRNA [NM_153607] | 10.66 |
| NM_015294 | Homo sapiens tripartite motif-containing 37 (TRIM37), transcript variant 1, mRNA [NM_015294] | 10.65 |
| NM_001690 | Homo sapiens ATPase, H+ transporting, lysosomal 70kDa, V1 subunit A (ATP6V1A), mRNA [NM_001690] | 10.64 |
| NM_017714 | Homo sapiens taspase, threonine aspartase, 1 (TASP1), mRNA [NM_017714] | 10.60 |
| NM_018046 | Homo sapiens angiogenic factor with G patch and FHA domains 1 (AGGF1), mRNA [NM_018046] | 10.59 |
| NM_018718 | Homo sapiens testis specific, 14 (TSGA14), mRNA [NM_018718] | 10.58 |
| NM_023927 | Homo sapiens GRAM domain containing 3 (GRAMD3), transcript variant 2, mRNA [NM_023927] | 10.57 |
| NM_080927 | Homo sapiens discoidin, CUB and LCCL domain containing 2 (DCBLD2), mRNA [NM_080927] | 10.55 |
| NM_012449 | Homo sapiens six transmembrane epithelial antigen of the prostate 1 (STEAP1), mRNA [NM_012449] | 10.54 |
| NM_018169 | Homo sapiens chromosome 12 open reading frame 35 (C12orf35), mRNA [NM_018169] | 10.51 |
| NM_005385 | Homo sapiens natural killer-tumor recognition sequence (NKTR), mRNA [NM_005385] | 10.50 |
| NM_020662 | Homo sapiens MRS2 magnesium homeostasis factor homolog (S. cerevisiae) (MRS2), mRNA [NM_020662] | 10.50 |
| NM_012194 | Homo sapiens chromosome 11 open reading frame 41 (C11orf41), mRNA [NM_012194] | 10.49 |
| NM_006364 | Homo sapiens Sec23 homolog A (S. cerevisiae) (SEC23A), mRNA [NM_006364] | 10.46 |
| NM_080632 | Homo sapiens UPF3 regulator of nonsense transcripts homolog B (yeast) (UPF3B), transcript variant 1, mRNA [NM_080632] | 10.46 |
| NM_001031723 | Homo sapiens DnaJ (Hsp40) homolog, subfamily B, member 14 (DNAJB14), transcript variant 1, mRNA [NM_001031723] | 10.45 |
| NM_019089 | Homo sapiens hairy and enhancer of split 2 (Drosophila) (HES2), mRNA [NM_019089] | 10.45 |
| NM_003641 | Homo sapiens interferon induced transmembrane protein 1 (9–27) (IFITM1), mRNA [NM_003641] | 10.41 |
| NM_018031 | Homo sapiens WD repeat domain 6 (WDR6), mRNA [NM_018031] | 10.38 |
| NM_138335 | Homo sapiens glucosamine-6-phosphate deaminase 2 (GNPDA2), mRNA [NM_138335] | 10.37 |
| NM_005154 | Homo sapiens ubiquitin specific peptidase 8 (USP8), transcript variant 1, mRNA [NM_005154] | 10.35 |
| NM_015475 | Homo sapiens family with sequence similarity 98, member A (FAM98A), mRNA [NM_015475] | 10.33 |
| NM_152284 | Homo sapiens chromatin modifying protein 4C (CHMP4C), mRNA [NM_152284] | 10.32 |
| NM_004888 | Homo sapiens ATPase, H+ transporting, lysosomal 13kDa, V1 subunit G1 (ATP6V1G1), mRNA [NM_004888] | 10.31 |

| | | |
|---|---|---|
| NM_000671 | Homo sapiens alcohol dehydrogenase 5 (class III), chi polypeptide (ADH5), mRNA [NM_000671] | 10.28 |
| NM_134442 | Homo sapiens cAMP responsive element binding protein 1 (CREB1), transcript variant B, mRNA [NM_134442] | 10.27 |
| NM_005988 | Homo sapiens small proline-rich protein 2A (SPRR2A), mRNA [NM_005988] | 10.25 |
| NM_001201329 | Homo sapiens protein phosphatase 1, regulatory (inhibitor) subunit 3B (PPP1R3B), transcript variant 1, mRNA [NM_001201329] | 10.20 |
| NM_033505 | Homo sapiens ethanolaminephosphotransferase 1 (CDP-ethanolamine-specific) (EPT1), mRNA [NM_033505] | 10.19 |
| NM_019050 | Homo sapiens ubiquitin specific peptidase 53 (USP53), mRNA [NM_019050] | 10.17 |
| NM_000899 | Homo sapiens KIT ligand (KITLG), transcript variant b, mRNA [NM_000899] | 10.16 |
| NM_017420 | Homo sapiens SIX homeobox 4 (SIX4), mRNA [NM_017420] | 10.13 |
| NM_005819 | Homo sapiens syntaxin 6 (STX6), mRNA [NM_005819] | 10.12 |
| NM_198401 | Homo sapiens ankyrin repeat domain 46 (ANKRD46), mRNA [NM_198401] | 10.10 |
| NM_021825 | Homo sapiens coiled-coil domain containing 90B (CCDC90B), mRNA [NM_021825] | 10.08 |
| NM_170743 | Homo sapiens interleukin 28 receptor, alpha (interferon, lambda receptor) (IL28RA), transcript variant 1, mRNA [NM_170743] | 10.05 |
| NM_001040619 | Homo sapiens activating transcription factor 3 (ATF3), transcript variant 4, mRNA [NM_001040619] | 10.05 |
| NM_015635 | Homo sapiens GTPase activating protein and VPS9 domains 1 (GAPVD1), mRNA [NM_015635] | 10.05 |
| NM_032564 | Homo sapiens diacylglycerol O-acyltransferase 2 (DGAT2), mRNA [NM_032564] | 10.04 |
| NM_003125 | Homo sapiens small proline-rich protein 1B (SPRR1B), mRNA [NM_003125] | 10.04 |
| NM_000021 | Homo sapiens presenilin 1 (PSEN1), transcript variant 1, mRNA [NM_000021] | 10.02 |
| NM_000314 | Homo sapiens phosphatase and tensin homolog (PTEN), mRNA [NM_000314] | 10.01 |
| NM_017837 | Homo sapiens phosphatidylinositol glycan anchor biosynthesis, class V (PIGV), mRNA [NM_017837] | 10.00 |
| NM_017763 | Homo sapiens ring finger protein 43 (RNF43), mRNA [NM_017763] | 10.00 |
| NM_003263 | Homo sapiens toll-like receptor 1 (TLR1), mRNA [NM_003263] | 9.98 |
| NM_001681 | Homo sapiens ATPase, Ca++ transporting, cardiac muscle, slow twitch 2 (ATP2A2), transcript variant 2, mRNA [NM_001681] | 9.97 |
| NM_176895 | Homo sapiens phosphatidic acid phosphatase type 2A (PPAP2A), transcript variant 2, mRNA [NM_176895] | 9.96 |
| NM_002534 | Homo sapiens 2’,5′-oligoadenylate synthetase 1, 40/46kDa (OAS1), transcript variant 2, mRNA [NM_002534] | 9.95 |

| | | |
|---|---|---|
| NM_138578 | Homo sapiens BCL2-like 1 (BCL2L1), nuclear gene encoding mitochondrial protein, transcript variant 1, mRNA [NM_138578] | 9.94 |
| NM_001613 | Homo sapiens actin, alpha 2, smooth muscle, aorta (ACTA2), transcript variant 2, mRNA [NM_001613] | 9.94 |
| NM_001039690 | Homo sapiens CTF8, chromosome transmission fidelity factor 8 homolog (S. cerevisiae) (CHTF8), transcript variant 1, mRNA [NM_001039690] | 9.93 |
| NM_001002010 | Homo sapiens 5′-nucleotidase, cytosolic III (NT5C3), transcript variant 1, mRNA [NM_001002010] | 9.92 |
| NM_002999 | Homo sapiens syndecan 4 (SDC4), mRNA [NM_002999] | 9.92 |
| NM_172071 | Homo sapiens ring finger and CCCH-type domains 1 (RC3H1), mRNA [NM_172071] | 9.90 |
| NM_016232 | Homo sapiens interleukin 1 receptor-like 1 (IL1RL1), transcript variant 1, mRNA [NM_016232] | 9.90 |
| NM_022873 | Homo sapiens interferon, alpha-inducible protein 6 (IFI6), transcript variant 3, mRNA [NM_022873] | 9.89 |
| NM_032860 | Homo sapiens LTV1 homolog (S. cerevisiae) (LTV1), mRNA [NM_032860] | 9.85 |
| NM_021229 | Homo sapiens netrin 4 (NTN4), mRNA [NM_021229] | 9.84 |
| NM_024523 | Homo sapiens GRIP and coiled-coil domain containing 1 (GCC1), mRNA [NM_024523] | 9.83 |
| NM_030627 | Homo sapiens cytoplasmic polyadenylation element binding protein 4 (CPEB4), mRNA [NM_030627] | 9.82 |
| NM_032924 | Homo sapiens zinc finger protein 3 (ZNF3), transcript variant 2, mRNA [NM_032924] | 9.82 |
| NM_018323 | Homo sapiens phosphatidylinositol 4-kinase type 2 beta (PI4K2B), mRNA [NM_018323] | 9.82 |
| NM_000153 | Homo sapiens galactosylceramidase (GALC), transcript variant 1, mRNA [NM_000153] | 9.82 |
| NM_014071 | Homo sapiens nuclear receptor coactivator 6 (NCOA6), mRNA [NM_014071] | 9.82 |
| NM_002906 | Homo sapiens radixin (RDX), mRNA [NM_002906] | 9.81 |
| NM_004186 | Homo sapiens sema domain, immunoglobulin domain (Ig), short basic domain, secreted, (semaphorin) 3F (SEMA3F), mRNA [NM_004186] | 9.80 |
| NM_004747 | Homo sapiens discs, large homolog 5 (Drosophila) (DLG5), mRNA [NM_004747] | 9.80 |
| NM_014824 | Homo sapiens FCH and double SH3 domains 2 (FCHSD2), mRNA [NM_014824] | 9.80 |
| NM_004925 | Homo sapiens aquaporin 3 (Gill blood group) (AQP3), mRNA [NM_004925] | 9.78 |
| NM_004598 | Homo sapiens sparc/osteonectin, cwcv and kazal-like domains proteoglycan (testican) 1 (SPOCK1), mRNA [NM_004598] | 9.78 |
| NM_014294 | Homo sapiens translocation associated membrane protein 1 (TRAM1), mRNA [NM_014294] | 9.77 |
| NM_012086 | Homo sapiens general transcription factor IIIC, polypeptide 3, 102kDa (GTF3C3), mRNA [NM_012086] | 9.77 |

| | | |
|---|---|---|
| NM_001031695 | Homo sapiens RNA binding protein, fox-1 homolog (C. elegans) 2 (RBFOX2), transcript variant 1, mRNA [NM_001031695] | 9.73 |
| NM_006890 | Homo sapiens carcinoembryonic antigen-related cell adhesion molecule 7 (CEACAM7), mRNA [NM_006890] | 9.72 |
| NM_001353 | Homo sapiens aldo-keto reductase family 1, member C1 (dihydrodiol dehydrogenase 1; 20-alpha (3-alpha)-hydroxysteroid dehydrogenase) (AKR1C1), mRNA [NM_001353] | 9.71 |
| NM_030918 | Homo sapiens sorting nexin family member 27 (SNX27), mRNA [NM_030918] | 9.70 |
| NM_145254 | Homo sapiens transmembrane protein 170A (TMEM170A), mRNA [NM_145254] | 9.69 |
| NM_015483 | Homo sapiens kelch repeat and BTB (POZ) domain containing 2 (KBTBD2), mRNA [NM_015483] | 9.69 |
| NM_003972 | Homo sapiens BTAF1 RNA polymerase II, B-TFIID transcription factor-associated, 170kDa (Mot1 homolog, S. cerevisiae) (BTAF1), mRNA [NM_003972] | 9.68 |
| NM_004627 | Homo sapiens tryptophan rich basic protein (WRB), transcript variant 1, mRNA [NM_004627] | 9.68 |
| NM_080750 | Homo sapiens DPH3, KTI11 homolog (S. cerevisiae) pseudogene 1 (DPH3P1), mRNA [NM_080750] | 9.66 |
| NM_017878 | Homo sapiens HRAS-like suppressor 2 (HRASLS2), mRNA [NM_017878] | 9.65 |
| NM_024616 | Homo sapiens chromosome 3 open reading frame 52 (C3orf52), transcript variant 2, mRNA [NM_024616] | 9.65 |
| NM_006080 | Homo sapiens sema domain, immunoglobulin domain (Ig), short basic domain, secreted, (semaphorin) 3A (SEMA3A), mRNA [NM_006080] | 9.64 |
| NM_005486 | Homo sapiens target of myb1 (chicken)-like 1 (TOM1L1), mRNA [NM_005486] | 9.62 |
| NM_001263 | Homo sapiens CDP-diacylglycerol synthase (phosphatidate cytidylyltransferase) 1 (CDS1), mRNA [NM_001263] | 9.61 |
| NM_004177 | Homo sapiens syntaxin 3 (STX3), transcript variant 1, mRNA [NM_004177] | 9.61 |
| NM_001105570 | Homo sapiens nudix (nucleoside diphosphate linked moiety X)-type motif 19 (NUDT19), nuclear gene encoding mitochondrial protein, mRNA [NM_001105570] | 9.60 |
| NM_003417 | Homo sapiens zinc finger protein 264 (ZNF264), mRNA [NM_003417] | 9.60 |
| NM_006820 | Homo sapiens interferon-induced protein 44-like (IFI44L), mRNA [NM_006820] | 9.59 |
| NM_183013 | Homo sapiens cAMP responsive element modulator (CREM), transcript variant 19, mRNA [NM_183013] | 9.59 |
| NM_016586 | Homo sapiens MAP3K12 binding inhibitory protein 1 (MBIP), transcript variant 1, mRNA [NM_016586] | 9.57 |
| NM_001412 | Homo sapiens eukaryotic translation initiation factor 1A, X-linked (EIF1AX), mRNA [NM_001412] | 9.56 |
| NM_018710 | Homo sapiens transmembrane protein 55A (TMEM55A), mRNA [NM_018710] | 9.56 |

| | | |
|---|---|---|
| NM_032199 | Homo sapiens AT rich interactive domain 5B (MRF1-like) (ARID5B), mRNA [NM_032199] | 9.56 |
| NM_001079839 | Homo sapiens OCIA domain containing 1 (OCIAD1), transcript variant 2, mRNA [NM_001079839] | 9.55 |
| NM_004226 | Homo sapiens serine/threonine kinase 17b (STK17B), mRNA [NM_004226] | 9.55 |
| NM_000640 | Homo sapiens interleukin 13 receptor, alpha 2 (IL13RA2), mRNA [NM_000640] | 9.53 |
| NM_024860 | Homo sapiens SET domain containing 6 (SETD6), transcript variant 2, mRNA [NM_024860] | 9.52 |
| NM_022767 | Homo sapiens apoptosis enhancing nuclease (AEN), mRNA [NM_022767] | 9.52 |
| NM_002294 | Homo sapiens lysosomal-associated membrane protein 2 (LAMP2), transcript variant A, mRNA [NM_002294] | 9.51 |
| NM_016082 | Homo sapiens CDK5 regulatory subunit associated protein 1 (CDK5RAP1), transcript variant 2, mRNA [NM_016082] | 9.50 |
| NM_004417 | Homo sapiens dual specificity phosphatase 1 (DUSP1), mRNA [NM_004417] | 9.50 |
| NM_017489 | Homo sapiens telomeric repeat binding factor (NIMA-interacting) 1 (TERF1), transcript variant 1, mRNA [NM_017489] | 9.49 |
| NM_003566 | Homo sapiens early endosome antigen 1 (EEA1), mRNA [NM_003566] | 9.47 |
| NM_003588 | Homo sapiens cullin 4B (CUL4B), transcript variant 1, mRNA [NM_003588] | 9.45 |
| NM_001912 | Homo sapiens cathepsin L1 (CTSL1), transcript variant 1, mRNA [NM_001912] | 9.45 |
| NM_006029 | Homo sapiens paraneoplastic antigen MA1 (PNMA1), mRNA [NM_006029] | 9.43 |
| NM_005966 | Homo sapiens NGFI-A binding protein 1 (EGR1 binding protein 1) (NAB1), mRNA [NM_005966] | 9.42 |
| NM_018051 | Homo sapiens WD repeat domain 60 (WDR60), mRNA [NM_018051] | 9.41 |
| NM_001551 | Homo sapiens immunoglobulin (CD79A) binding protein 1 (IGBP1), mRNA [NM_001551] | 9.41 |
| NM_017913 | Homo sapiens cell division cycle 37 homolog (S. cerevisiae)-like 1 (CDC37L1), mRNA [NM_017913] | 9.40 |
| NM_002703 | Homo sapiens phosphoribosyl pyrophosphate amidotransferase (PPAT), mRNA [NM_002703] | 9.40 |
| NM_013943 | Homo sapiens chloride intracellular channel 4 (CLIC4), nuclear gene encoding mitochondrial protein, mRNA [NM_013943] | 9.39 |
| NM_002853 | Homo sapiens RAD1 homolog (S. pombe) (RAD1), transcript variant 1, mRNA [NM_002853] | 9.37 |
| NM_001001937 | Homo sapiens ATP synthase, H+ transporting, mitochondrial F1 complex, alpha subunit 1, cardiac muscle (ATP5A1), nuclear gene encoding mitochondrial protein, transcript variant 1, mRNA [NM_001001937] | 9.34 |
| NM_014089 | Homo sapiens nucleoporin like 1 (NUPL1), transcript variant 1, mRNA [NM_014089] | 9.33 |

| | | |
|---|---|---|
| NM_006047 | Homo sapiens RNA binding motif protein 12 (RBM12), transcript variant 1, mRNA [NM_006047] | 9.32 |
| NM_173567 | Homo sapiens epoxide hydrolase 4 (EPHX4), mRNA [NM_173567] | 9.32 |
| NM_024690 | Homo sapiens mucin 16, cell surface associated (MUC16), mRNA [NM_024690] | 9.32 |
| NM_001102426 | Homo sapiens TBC1 domain family, member 8 (with GRAM domain) (TBC1D8), mRNA [NM_001102426] | 9.32 |
| NM_130837 | Homo sapiens optic atrophy 1 (autosomal dominant) (OPA1), nuclear gene encoding mitochondrial protein, transcript variant 8, mRNA [NM_130837] | 9.30 |
| NM_018290 | Homo sapiens phosphoglucomutase 2 (PGM2), mRNA [NM_018290] | 9.30 |
| NM_032194 | Homo sapiens ribosome production factor 2 homolog (S. cerevisiae) (RPF2), mRNA [NM_032194] | 9.30 |
| NM_032783 | Homo sapiens carbonyl reductase 4 (CBR4), mRNA [NM_032783] | 9.30 |
| NM_001338 | Homo sapiens coxsackie virus and adenovirus receptor (CXADR), mRNA [NM_001338] | 9.30 |
| NM_001040441 | Homo sapiens zinc finger and BTB domain containing 8A (ZBTB8A), mRNA [NM_001040441] | 9.29 |
| NM_002153 | Homo sapiens hydroxysteroid (17-beta) dehydrogenase 2 (HSD17B2), mRNA [NM_002153] | 9.29 |
| NM_018638 | Homo sapiens ethanolamine kinase 1 (ETNK1), transcript variant 1, mRNA [NM_018638] | 9.28 |
| NM_014415 | Homo sapiens zinc finger and BTB domain containing 11 (ZBTB11), mRNA [NM_014415] | 9.27 |
| NM_002114 | Homo sapiens human immunodeficiency virus type I enhancer binding protein 1 (HIVEP1), mRNA [NM_002114] | 9.26 |
| NM_004290 | Homo sapiens ring finger protein 14 (RNF14), transcript variant 1, mRNA [NM_004290] | 9.26 |
| NM_203462 | Homo sapiens Morf4 family associated protein 1-like 1 (MRFAP1L1), mRNA [NM_203462] | 9.25 |
| NM_018117 | Homo sapiens WD repeat domain 11 (WDR11), mRNA [NM_018117] | 9.25 |
| NM_015515 | Homo sapiens keratin 23 (histone deacetylase inducible) (KRT23), mRNA [NM_015515] | 9.23 |
| NM_021129 | Homo sapiens pyrophosphatase (inorganic) 1 (PPA1), mRNA [NM_021129] | 9.22 |
| NM_024770 | Homo sapiens methyltransferase like 8 (METTL8), mRNA [NM_024770] | 9.22 |
| NM_003844 | Homo sapiens tumor necrosis factor receptor superfamily, member 10a (TNFRSF10A), mRNA [NM_003844] | 9.19 |
| NM_024830 | Homo sapiens lysophosphatidylcholine acyltransferase 1 (LPCAT1), mRNA [NM_024830] | 9.18 |
| NM_001098621 | Homo sapiens chromosome 14 open reading frame 109 (C14orf109), transcript variant 1, mRNA [NM_001098621] | 9.17 |
| NM_006357 | Homo sapiens ubiquitin-conjugating enzyme E2E 3 (UBC4/5 homolog, yeast) (UBE2E3), transcript variant 1, mRNA [NM_006357] | 9.17 |

| | | |
|---|---|---|
| NM_030963 | Homo sapiens ring finger protein 146 (RNF146), mRNA [NM_030963] | 9.16 |
| NM_019006 | Homo sapiens zinc finger, AN1-type domain 6 (ZFAND6), mRNA [NM_019006] | 9.16 |
| NM_144567 | Homo sapiens angel homolog 2 (Drosophila) (ANGEL2), mRNA [NM_144567] | 9.16 |
| NM_138444 | Homo sapiens potassium channel tetramerisation domain containing 12 (KCTD12), mRNA [NM_138444] | 9.15 |
| NM_133493 | Homo sapiens CD109 molecule (CD109), transcript variant 1, mRNA [NM_133493] | 9.14 |
| NM_138461 | Homo sapiens transmembrane 4 L six family member 19 (TM4SF19), mRNA [NM_138461] | 9.13 |
| NM_014947 | Homo sapiens forkhead box J3 (FOXJ3), transcript variant 1, mRNA [NM_014947] | 9.12 |
| NM_014614 | Homo sapiens proteasome (prosome, macropain) activator subunit 4 (PSME4), mRNA [NM_014614] | 9.11 |
| NM_001099270 | Homo sapiens zinc finger and BTB domain containing 34 (ZBTB34), mRNA [NM_001099270] | 9.11 |
| NM_001098398 | Homo sapiens coatomer protein complex, subunit alpha (COPA), transcript variant 1, mRNA [NM_001098398] | 9.11 |
| NM_006915 | Homo sapiens retinitis pigmentosa 2 (X-linked recessive) (RP2), mRNA [NM_006915] | 9.10 |
| NM_054016 | Homo sapiens serine/arginine-rich splicing factor 10 (SRSF10), transcript variant 2, mRNA [NM_054016] | 9.08 |
| NM_018122 | Homo sapiens aspartyl-tRNA synthetase 2, mitochondrial (DARS2), nuclear gene encoding mitochondrial protein, mRNA [NM_018122] | 9.08 |
| NM_022496 | Homo sapiens ARP6 actin-related protein 6 homolog (yeast) (ACTR6), mRNA [NM_022496] | 9.08 |
| NM_016275 | Homo sapiens selenoprotein T (SELT), mRNA [NM_016275] | 9.06 |
| NM_017761 | Homo sapiens proline-rich nuclear receptor coactivator 2 (PNRC2), mRNA [NM_017761] | 9.05 |
| NM_152407 | Homo sapiens GrpE-like 2, mitochondrial (E. coli) (GRPEL2), nuclear gene encoding mitochondrial protein, mRNA [NM_152407] | 9.04 |
| NM_016354 | Homo sapiens solute carrier organic anion transporter family, member 4A1 (SLCO4A1), mRNA [NM_016354] | 9.04 |
| NM_012091 | Homo sapiens adenosine deaminase, tRNA-specific 1 (ADAT1), transcript variant 1, mRNA [NM_012091] | 9.04 |
| NM_025195 | Homo sapiens tribbles homolog 1 (Drosophila) (TRIB1), mRNA [NM_025195] | 9.04 |
| NM_032466 | Homo sapiens aspartate beta-hydroxylase (ASPH), transcript variant 3, mRNA [NM_032466] | 9.03 |
| NM_080618 | Homo sapiens CCCTC-binding factor (zinc finger protein)-like (CTCFL), mRNA [NM_080618] | 9.02 |
| NM_014709 | Homo sapiens ubiquitin specific peptidase 34 (USP34), mRNA [NM_014709] | 9.02 |
| NM_080801 | Homo sapiens collagen, type XIII, alpha 1 (COL13A1), transcript variant 5, mRNA [NM_080801] | 9.01 |

| | | |
|---|---|---|
| NM_000628 | Homo sapiens interleukin 10 receptor, beta (IL10RB), mRNA [NM_000628] | 9.01 |
| NM_024911 | Homo sapiens wntless homolog (Drosophila) (WLS), transcript variant 1, mRNA [NM_024911] | 9.00 |
| NM_001190839 | Homo sapiens matrix Gla protein (MGP), transcript variant 1, mRNA [NM_001190839] | 9.00 |
| NM_006947 | Homo sapiens signal recognition particle 72kDa (SRP72), mRNA [NM_006947] | 8.98 |
| NM_007039 | Homo sapiens protein tyrosine phosphatase, non-receptor type 21 (PTPN21), mRNA [NM_007039] | 8.97 |
| NM_182532 | Homo sapiens transmembrane protein 61 (TMEM61), mRNA [NM_182532] | 8.94 |
| NM_016258 | Homo sapiens YTH domain family, member 2 (YTHDF2), transcript variant 1, mRNA [NM_016258] | 8.94 |
| NM_005455 | Homo sapiens zinc finger, RAN-binding domain containing 2 (ZRANB2), transcript variant 2, mRNA [NM_005455] | 8.94 |
| NM_000240 | Homo sapiens monoamine oxidase A (MAOA), nuclear gene encoding mitochondrial protein, mRNA [NM_000240] | 8.92 |
| NM_002598 | Homo sapiens programmed cell death 2 (PDCD2), transcript variant 1, mRNA [NM_002598] | 8.92 |
| NM_053023 | Homo sapiens zinc finger protein 91 homolog (mouse) (ZFP91), transcript variant 1, mRNA [NM_053023] | 8.91 |
| NM_019015 | Homo sapiens chondroitin polymerizing factor 2 (CHPF2), mRNA [NM_019015] | 8.91 |
| NM_004728 | Homo sapiens DEAD (Asp-Glu-Ala-Asp) box polypeptide 21 (DDX21), mRNA [NM_004728] | 8.91 |
| NM_021925 | Homo sapiens chromosome 2 open reading frame 43 (C2orf43), mRNA [NM_021925] | 8.91 |
| NM_012158 | Homo sapiens F-box and leucine-rich repeat protein 3 (FBXL3), mRNA [NM_012158] | 8.91 |
| NM_138423 | Homo sapiens cancer susceptibility candidate 4 (CASC4), transcript variant 1, mRNA [NM_138423] | 8.91 |
| NM_024604 | Homo sapiens RNA polymerase II associated protein 3 (RPAP3), transcript variant 1, mRNA [NM_024604] | 8.89 |
| NM_173834 | Homo sapiens Yip1 domain family, member 6 (YIPF6), transcript variant A, mRNA [NM_173834] | 8.89 |
| NM_005499 | Homo sapiens ubiquitin-like modifier activating enzyme 2 (UBA2), mRNA [NM_005499] | 8.89 |
| NM_012424 | Homo sapiens ribosomal protein S6 kinase, 52kDa, polypeptide 1 (RPS6KC1), transcript variant 1, mRNA [NM_012424] | 8.85 |
| NM_001539 | Homo sapiens DnaJ (Hsp40) homolog, subfamily A, member 1 (DNAJA1), mRNA [NM_001539] | 8.83 |
| NM_004663 | Homo sapiens RAB11A, member RAS oncogene family (RAB11A), mRNA [NM_004663] | 8.83 |
| NM_182691 | Homo sapiens SRSF protein kinase 2 (SRPK2), transcript variant 2, mRNA [NM_182691] | 8.83 |

| | | |
|---|---|---|
| NM_021804 | Homo sapiens angiotensin I converting enzyme (peptidyl-dipeptidase A) 2 (ACE2), mRNA [NM_021804] | 8.83 |
| NM_003713 | Homo sapiens phosphatidic acid phosphatase type 2B (PPAP2B), mRNA [NM_003713] | 8.82 |
| NM_001001992 | Homo sapiens ubiquitin specific peptidase 16 (USP16), transcript variant 2, mRNA [NM_001001992] | 8.81 |
| NM_002658 | Homo sapiens plasminogen activator, urokinase (PLAU), transcript variant 1, mRNA [NM_002658] | 8.81 |
| NM_012415 | Homo sapiens RAD54 homolog B (S. cerevisiae) (RAD54B), mRNA [NM_012415] | 8.79 |
| NM_015001 | Homo sapiens spen homolog, transcriptional regulator (Drosophila) (SPEN), mRNA [NM_015001] | 8.78 |
| NM_018480 | Homo sapiens transmembrane protein 126B (TMEM126B), transcript variant 1, mRNA [NM_018480] | 8.78 |
| NM_033550 | Homo sapiens TP53 regulating kinase (TP53RK), mRNA [NM_033550] | 8.77 |
| NM_001001552 | Homo sapiens LEM domain containing 1 (LEMD1), transcript variant 3, mRNA [NM_001001552] | 8.76 |
| NM_004155 | Homo sapiens serpin peptidase inhibitor, clade B (ovalbumin), member 9 (SERPINB9), mRNA [NM_004155] | 8.76 |
| NM_014758 | Homo sapiens sorting nexin 19 (SNX19), mRNA [NM_014758] | 8.76 |
| NM_024101 | Homo sapiens melanophilin (MLPH), transcript variant 1, mRNA [NM_024101] | 8.73 |
| NM_001136007 | Homo sapiens FXYD domain containing ion transport regulator 3 (FXYD3), transcript variant 3, mRNA [NM_001136007] | 8.73 |
| NM_005450 | Homo sapiens noggin (NOG), mRNA [NM_005450] | 8.72 |
| NM_145168 | Homo sapiens short chain dehydrogenase/reductase family 42E, member 1 (SDR42E1), mRNA [NM_145168] | 8.72 |
| NM_003507 | Homo sapiens frizzled homolog 7 (Drosophila) (FZD7), mRNA [NM_003507] | 8.71 |
| NM_002354 | Homo sapiens epithelial cell adhesion molecule (EPCAM), mRNA [NM_002354] | 8.71 |
| NM_198041 | Homo sapiens nudix (nucleoside diphosphate linked moiety X)-type motif 6 (NUDT6), transcript variant 2, mRNA [NM_198041] | 8.71 |
| NM_001037165 | Homo sapiens forkhead box K1 (FOXK1), mRNA [NM_001037165] | 8.70 |
| NM_004510 | Homo sapiens SP110 nuclear body protein (SP110), transcript variant b, mRNA [NM_004510] | 8.70 |
| NM_012155 | Homo sapiens echinoderm microtubule associated protein like 2 (EML2), transcript variant 2, mRNA [NM_012155] | 8.68 |
| NM_152622 | Homo sapiens mesoderm induction early response 1, family member 3 (MIER3), mRNA [NM_152622] | 8.68 |
| NM_014892 | Homo sapiens SR-related CTD-associated factor 8 (SCAF8), mRNA [NM_014892] | 8.67 |
| NM_058004 | Homo sapiens phosphatidylinositol 4-kinase, catalytic, alpha (PI4KA), transcript variant 2, mRNA [NM_058004] | 8.66 |

| | | |
|---|---|---|
| NM_014705 | Homo sapiens dedicator of cytokinesis 4 (DOCK4), mRNA [NM_014705] | 8.66 |
| NM_021132 | Homo sapiens protein phosphatase 3, catalytic subunit, beta isozyme (PPP3CB), transcript variant 2, mRNA [NM_021132] | 8.65 |
| NM_015306 | Homo sapiens ubiquitin specific peptidase 24 (USP24), mRNA [NM_015306] | 8.65 |
| NM_004972 | Homo sapiens Janus kinase 2 (JAK2), mRNA [NM_004972] | 8.64 |
| NM_016618 | Homo sapiens lysine-rich coiled-coil 1 (KRCC1), mRNA [NM_016618] | 8.63 |
| NM_004929 | Homo sapiens calbindin 1, 28kDa (CALB1), mRNA [NM_004929] | 8.63 |
| NM_014247 | Homo sapiens Rap guanine nucleotide exchange factor (GEF) 2 (RAPGEF2), mRNA [NM_014247] | 8.60 |
| NM_006618 | Homo sapiens lysine (K)-specific demethylase 5B (KDM5B), mRNA [NM_006618] | 8.60 |
| NM_033017 | Homo sapiens tripartite motif-containing 4 (TRIM4), transcript variant alpha, mRNA [NM_033017] | 8.60 |
| NM_024956 | Homo sapiens transmembrane protein 62 (TMEM62), mRNA [NM_024956] | 8.59 |
| NM_152791 | Homo sapiens zinc finger protein 555 (ZNF555), transcript variant 1, mRNA [NM_152791] | 8.58 |
| NM_020336 | Homo sapiens Ral GTPase activating protein, beta subunit (non-catalytic) (RALGAPB), mRNA [NM_020336] | 8.57 |
| NM_182643 | Homo sapiens deleted in liver cancer 1 (DLC1), transcript variant 1, mRNA [NM_182643] | 8.55 |
| NM_153608 | Homo sapiens zinc finger protein 114 (ZNF114), mRNA [NM_153608] | 8.54 |
| NM_001044723 | Homo sapiens casein kinase 1, gamma 3 (CSNK1G3), transcript variant 4, mRNA [NM_001044723] | 8.54 |
| NM_022753 | Homo sapiens S100P binding protein (S100PBP), transcript variant 1, mRNA [NM_022753] | 8.53 |
| NM_020448 | Homo sapiens NIPA-like domain containing 3 (NIPAL3), mRNA [NM_020448] | 8.52 |
| NM_031208 | Homo sapiens fumarylacetoacetate hydrolase domain containing 1 (FAHD1), nuclear gene encoding mitochondrial protein, transcript variant 2, mRNA [NM_031208] | 8.50 |
| NM_001195220 | Homo sapiens zinc finger family member 783 (ZNF783), mRNA [NM_001195220] | 8.50 |
| NM_014285 | Homo sapiens exosome component 2 (EXOSC2), mRNA [NM_014285] | 8.50 |
| NM_007048 | Homo sapiens butyrophilin, subfamily 3, member A1 (BTN3A1), transcript variant 1, mRNA [NM_007048] | 8.49 |
| NM_015161 | Homo sapiens ADP-ribosylation factor-like 6 interacting protein 1 (ARL6IP1), mRNA [NM_015161] | 8.48 |
| NM_031229 | Homo sapiens RanBP-type and C3HC4-type zinc finger containing 1 (RBCK1), transcript variant 2, mRNA [NM_031229] | 8.47 |
| NM_001039091 | Homo sapiens phosphoribosyl pyrophosphate synthetase 2 (PRPS2), transcript variant 1, mRNA [NM_001039091] | 8.47 |

| | | |
|---|---|---|
| NM_032139 | Homo sapiens ankyrin repeat domain 27 (VPS9 domain) (ANKRD27), mRNA [NM_032139] | 8.47 |
| NM_017631 | Homo sapiens DEAD (Asp-Glu-Ala-Asp) box polypeptide 60 (DDX60), mRNA [NM_017631] | 8.46 |
| NM_014951 | Homo sapiens zinc finger protein 365 (ZNF365), transcript variant A, mRNA [NM_014951] | 8.46 |
| NM_001077710 | Homo sapiens family with sequence similarity 110, member C (FAM110C), mRNA [NM_001077710] | 8.45 |
| NM_000532 | Homo sapiens propionyl CoA carboxylase, beta polypeptide (PCCB), nuclear gene encoding mitochondrial protein, transcript variant 1, mRNA [NM_000532] | 8.44 |
| NM_002946 | Homo sapiens replication protein A2, 32kDa (RPA2), mRNA [NM_002946] | 8.42 |
| NM_020632 | Homo sapiens ATPase, H+ transporting, lysosomal V0 subunit a4 (ATP6V0A4), transcript variant 1, mRNA [NM_020632] | 8.41 |
| NM_000426 | Homo sapiens laminin, alpha 2 (LAMA2), transcript variant 1, mRNA [NM_000426] | 8.41 |
| NM_019012 | Homo sapiens pleckstrin homology domain containing, family A member 5 (PLEKHA5), transcript variant 1, mRNA [NM_019012] | 8.41 |
| NM_012062 | Homo sapiens dynamin 1-like (DNM1L), transcript variant 1, mRNA [NM_012062] | 8.41 |
| NM_198147 | Homo sapiens abhydrolase domain containing 15 (ABHD15), mRNA [NM_198147] | 8.41 |
| NM_015350 | Homo sapiens leucine rich repeat containing 8 family, member B (LRRC8B), transcript variant 1, mRNA [NM_015350] | 8.40 |
| AF001893 | gb\|Human MEN1 region clone epsilon/beta mRNA, 3' fragment. [AF001893] | 8.40 |
| NM_024617 | Homo sapiens zinc finger, CCHC domain containing 6 (ZCCHC6), transcript variant 1, mRNA [NM_024617] | 8.40 |
| NM_177938 | Homo sapiens prolyl 4-hydroxylase, transmembrane (endoplasmic reticulum) (P4HTM), transcript variant 3, mRNA [NM_177938] | 8.39 |
| NM_015061 | Homo sapiens lysine (K)-specific demethylase 4C (KDM4C), transcript variant 1, mRNA [NM_015061] | 8.39 |
| NM_175866 | Homo sapiens U2AF homology motif (UHM) kinase 1 (UHMK1), transcript variant 1, mRNA [NM_175866] | 8.39 |
| NM_022909 | Homo sapiens centromere protein H (CENPH), mRNA [NM_022909] | 8.38 |
| NM_002271 | Homo sapiens importin 5 (IPO5), mRNA [NM_002271] | 8.38 |
| NM_004633 | Homo sapiens interleukin 1 receptor, type II (IL1R2), transcript variant 1, mRNA [NM_004633] | 8.38 |
| NM_002601 | Homo sapiens phosphodiesterase 6D, cGMP-specific, rod, delta (PDE6D), mRNA [NM_002601] | 8.38 |
| NM_022334 | Homo sapiens integrin beta 1 binding protein 1 (ITGB1BP1), transcript variant 2, mRNA [NM_022334] | 8.38 |
| NM_016248 | Homo sapiens A kinase (PRKA) anchor protein 11 (AKAP11), mRNA [NM_016248] | 8.37 |

| | | |
|---|---|---|
| NM_022149 | Homo sapiens melanoma antigen family F, 1 (MAGEF1), mRNA [NM_022149] | 8.37 |
| NM_003222 | Homo sapiens transcription factor AP-2 gamma (activating enhancer binding protein 2 gamma) (TFAP2C), mRNA [NM_003222] | 8.37 |
| NM_018656 | Homo sapiens solute carrier family 35, member E3 (SLC35E3), mRNA [NM_018656] | 8.36 |
| NM_017860 | Homo sapiens chromosome 1 open reading frame 56 (C1orf56), mRNA [NM_017860] | 8.36 |
| NM_005063 | Homo sapiens stearoyl-CoA desaturase (delta-9-desaturase) (SCD), mRNA [NM_005063] | 8.36 |
| NM_006806 | Homo sapiens BTG family, member 3 (BTG3), transcript variant 2, mRNA [NM_006806] | 8.36 |
| NM_206907 | Homo sapiens protein kinase, AMP-activated, alpha 1 catalytic subunit (PRKAA1), transcript variant 2, mRNA [NM_206907] | 8.35 |
| NM_020740 | Homo sapiens ankyrin repeat and FYVE domain containing 1 (ANKFY1), transcript variant 2, mRNA [NM_020740] | 8.34 |
| NM_030817 | Homo sapiens apolipoprotein L domain containing 1 (APOLD1), transcript variant 2, mRNA [NM_030817] | 8.34 |
| NM_015702 | Homo sapiens methylmalonic aciduria (cobalamin deficiency) cblD type, with homocystinuria (MMADHC), nuclear gene encoding mitochondrial protein, mRNA [NM_015702] | 8.34 |
| NM_153358 | Homo sapiens zinc finger protein 791 (ZNF791), mRNA [NM_153358] | 8.34 |
| NM_032181 | Homo sapiens family with sequence similarity 176, member A (FAM176A), transcript variant 2, mRNA [NM_032181] | 8.33 |
| NM_014715 | Homo sapiens Rho GTPase activating protein 32 (ARHGAP32), transcript variant 2, mRNA [NM_014715] | 8.33 |
| NM_001331 | Homo sapiens catenin (cadherin-associated protein), delta 1 (CTNND1), transcript variant 3, mRNA [NM_001331] | 8.31 |
| NM_173555 | Homo sapiens trypsin domain containing 1 (TYSND1), transcript variant 1, mRNA [NM_173555] | 8.31 |
| NM_020679 | Homo sapiens MIF4G domain containing (MIF4GD), mRNA [NM_020679] | 8.31 |
| NM_019594 | Homo sapiens leucine rich repeat containing 8 family, member A (LRRC8A), transcript variant 2, mRNA [NM_019594] | 8.31 |
| NM_020214 | Homo sapiens poly (ADP-ribose) polymerase family, member 6 (PARP6), mRNA [NM_020214] | 8.31 |
| NM_004289 | Homo sapiens nuclear factor (erythroid-derived 2)-like 3 (NFE2L3), mRNA [NM_004289] | 8.30 |
| NM_181787 | Homo sapiens dpy-19-like 4 (C. elegans) (DPY19L4), mRNA [NM_181787] | 8.29 |
| NM_139072 | Homo sapiens delta/notch-like EGF repeat containing (DNER), mRNA [NM_139072] | 8.29 |
| NM_017742 | Homo sapiens zinc finger, CCHC domain containing 2 (ZCCHC2), mRNA [NM_017742] | 8.28 |
| NM_014822 | Homo sapiens SEC24 family, member D (S. cerevisiae) (SEC24D), mRNA [NM_014822] | 8.28 |

| | | |
|---|---|---|
| NM_001145154 | Homo sapiens dynein, axonemal, heavy chain 14 (DNAH14), transcript variant 2, mRNA [NM_001145154] | 8.28 |
| NM_001145112 | Homo sapiens protein associated with topoisomerase II homolog 2 (yeast) (PATL2), mRNA [NM_001145112] | 8.27 |
| NM_145247 | Homo sapiens chromosome 10 open reading frame 78 (C10orf78), transcript variant 2, mRNA [NM_145247] | 8.27 |
| NM_012399 | Homo sapiens phosphatidylinositol transfer protein, beta (PITPNB), mRNA [NM_012399] | 8.26 |
| NM_005872 | Homo sapiens breast carcinoma amplified sequence 2 (BCAS2), mRNA [NM_005872] | 8.25 |
| NM_030647 | Homo sapiens jumonji C domain containing histone demethylase 1 homolog D (S. cerevisiae) (JHDM1D), mRNA [NM_030647] | 8.25 |
| NM_004779 | Homo sapiens CCR4-NOT transcription complex, subunit 8 (CNOT8), mRNA [NM_004779] | 8.24 |
| NM_006466 | Homo sapiens polymerase (RNA) III (DNA directed) polypeptide F, 39 kDa (POLR3F), mRNA [NM_006466] | 8.24 |
| NM_003463 | Homo sapiens protein tyrosine phosphatase type IVA, member 1 (PTP4A1), mRNA [NM_003463] | 8.23 |
| NM_001017402 | Homo sapiens laminin, beta 3 (LAMB3), transcript variant 2, mRNA [NM_001017402] | 8.23 |
| NM_172037 | Homo sapiens retinol dehydrogenase 10 (all-trans) (RDH10), mRNA [NM_172037] | 8.20 |
| XM_003119360 | PREDICTED: Homo sapiens HLA class I histocompatibility antigen, Cw-15 alpha chain-like, transcript variant 2 (LOC100507681), mRNA [XM_003119360] | 8.20 |
| NM_006888 | Homo sapiens calmodulin 1 (phosphorylase kinase, delta) (CALM1), transcript variant 1, mRNA [NM_006888] | 8.20 |
| NM_019029 | Homo sapiens carboxypeptidase, vitellogenic-like (CPVL), transcript variant 2, mRNA [NM_019029] | 8.20 |
| NM_005643 | Homo sapiens TAF11 RNA polymerase II, TATA box binding protein (TBP)-associated factor, 28kDa (TAF11), mRNA [NM_005643] | 8.19 |
| NM_018847 | Homo sapiens kelch-like 9 (Drosophila) (KLHL9), mRNA [NM_018847] | 8.19 |
| NM_004268 | Homo sapiens mediator complex subunit 17 (MED17), mRNA [NM_004268] | 8.19 |
| NM_003887 | Homo sapiens ArfGAP with SH3 domain, ankyrin repeat and PH domain 2 (ASAP2), transcript variant 1, mRNA [NM_003887] | 8.18 |
| NM_002064 | Homo sapiens glutaredoxin (thioltransferase) (GLRX), transcript variant 1, mRNA [NM_002064] | 8.18 |
| NM_001006683 | Homo sapiens spindlin family, member 2B (SPIN2B), transcript variant 3, mRNA [NM_001006683] | 8.18 |
| NM_173690 | Homo sapiens suppressor of cancer cell invasion (SCAI), transcript variant 1, mRNA [NM_173690] | 8.16 |
| NM_014445 | Homo sapiens stress-associated endoplasmic reticulum protein 1 (SERP1), mRNA [NM_014445] | 8.13 |
| NM_183049 | Homo sapiens thymosin-like 3 (TMSL3), mRNA [NM_183049] | 8.13 |

| | | |
|---|---|---|
| NM_016086 | Homo sapiens serine/threonine/tyrosine interacting-like 1 (STYXL1), mRNA [NM_016086] | 8.13 |
| NM_006074 | Homo sapiens tripartite motif-containing 22 (TRIM22), transcript variant 1, mRNA [NM_006074] | 8.12 |
| NM_018036 | Homo sapiens ATG2 autophagy related 2 homolog B (S. cerevisiae) (ATG2B), mRNA [NM_018036] | 8.11 |
| NM_173797 | Homo sapiens PAP associated domain containing 4 (PAPD4), transcript variant 3, mRNA [NM_173797] | 8.11 |
| NM_001135187 | Homo sapiens ArfGAP with FG repeats 1 (AGFG1), transcript variant 1, mRNA [NM_001135187] | 8.11 |
| NM_020755 | Homo sapiens serine incorporator 1 (SERINC1), mRNA [NM_020755] | 8.10 |
| NM_000286 | Homo sapiens peroxisomal biogenesis factor 12 (PEX12), mRNA [NM_000286] | 8.10 |
| NM_004457 | Homo sapiens acyl-CoA synthetase long-chain family member 3 (ACSL3), transcript variant 1, mRNA [NM_004457] | 8.10 |
| NM_005470 | Homo sapiens abl-interactor 1 (ABI1), transcript variant 1, mRNA [NM_005470] | 8.08 |
| NM_001001716 | Homo sapiens nuclear factor of kappa light polypeptide gene enhancer in B-cells inhibitor, beta (NFKBIB), transcript variant 2, mRNA [NM_001001716] | 8.08 |
| NM_004294 | Homo sapiens mitochondrial translational release factor 1 (MTRF1), nuclear gene encoding mitochondrial protein, mRNA [NM_004294] | 8.08 |
| NM_002033 | Homo sapiens fucosyltransferase 4 (alpha (1,3) fucosyltransferase, myeloid-specific) (FUT4), mRNA [NM_002033] | 8.08 |
| NM_019555 | Homo sapiens Rho guanine nucleotide exchange factor (GEF) 3 (ARHGEF3), transcript variant 3, mRNA [NM_019555] | 8.07 |
| NM_021970 | Homo sapiens MAPK scaffold protein 1 (MAPKSP1), transcript variant 1, mRNA [NM_021970] | 8.07 |
| NM_198129 | Homo sapiens laminin, alpha 3 (LAMA3), transcript variant 1, mRNA [NM_198129] | 8.07 |
| NM_004642 | Homo sapiens cyclin-dependent kinase 2 associated protein 1 (CDK2AP1), mRNA [NM_004642] | 8.07 |
| NM_018343 | Homo sapiens RIO kinase 2 (yeast) (RIOK2), transcript variant 1, mRNA [NM_018343] | 8.07 |
| NM_005476 | Homo sapiens glucosamine (UDP-N-acetyl)-2-epimerase/N-acetylmannosamine kinase (GNE), transcript variant 2, mRNA [NM_005476] | 8.07 |
| NM_017881 | Homo sapiens chromosome 9 open reading frame 95 (C9orf95), transcript variant 1, mRNA [NM_017881] | 8.06 |
| NM_015224 | Homo sapiens chromosome 3 open reading frame 63 (C3orf63), transcript variant 2, mRNA [NM_015224] | 8.06 |
| NM_022096 | Homo sapiens ankyrin repeat domain 5 (ANKRD5), transcript variant 1, mRNA [NM_022096] | 8.06 |
| NM_020147 | Homo sapiens THAP domain containing 10 (THAP10), mRNA [NM_020147] | 8.05 |

| | | |
|---|---|---|
| NM_002577 | Homo sapiens p21 protein (Cdc42/Rac)-activated kinase 2 (PAK2), mRNA [NM_002577] | 8.05 |
| NM_003900 | Homo sapiens sequestosome 1 (SQSTM1), transcript variant 1, mRNA [NM_003900] | 8.04 |
| NM_030919 | Homo sapiens family with sequence similarity 83, member D (FAM83D), mRNA [NM_030919] | 8.04 |
| NM_001001555 | Homo sapiens growth factor receptor-bound protein 10 (GRB10), transcript variant 4, mRNA [NM_001001555] | 8.02 |
| NM_153810 | Homo sapiens chromosome 10 open reading frame 46 (C10orf46), mRNA [NM_153810] | 8.02 |
| NM_000100 | Homo sapiens cystatin B (stefin B) (CSTB), mRNA [NM_000100] | 8.01 |
| NM_006670 | Homo sapiens trophoblast glycoprotein (TPBG), transcript variant 1, mRNA [NM_006670] | 8.01 |
| NM_018420 | Homo sapiens solute carrier family 22, member 15 (SLC22A15), mRNA [NM_018420] | 8.00 |
| NM_134470 | Homo sapiens interleukin 1 receptor accessory protein (IL1RAP), transcript variant 2, mRNA [NM_134470] | 8.00 |
| NM_014230 | Homo sapiens signal recognition particle 68kDa (SRP68), mRNA [NM_014230] | 8.00 |
| NM_002485 | Homo sapiens nibrin (NBN), mRNA [NM_002485] | 8.00 |
